# TGF-β-dependent lymphoid tissue residency of stem-like T cells limits response to tumor vaccine

**DOI:** 10.1038/s41467-022-33768-x

**Published:** 2022-10-13

**Authors:** Guo Li, Saranya Srinivasan, Liwen Wang, Chaoyu Ma, Kai Guo, Wenhao Xiao, Wei Liao, Shruti Mishra, Xin Zhang, Yuanzheng Qiu, Qianjin Lu, Yong Liu, Nu Zhang

**Affiliations:** 1 Department of Microbiology, Immunology and Molecular Genetics, Long School of Medicine, University of Texas Health Science Center at San Antonio, San Antonio, TX 78229 USA; 2 Department of Otolaryngology Head and Neck Surgery, Xiangya Hospital, Central South University, 87 Xiangya Road, Changsha, Hunan 410008 China; 3 Otolaryngology Major Disease Research Key Laboratory of Hunan Province, Xiangya Hospital, Central South University, Changsha, Hunan 410008 China; 4 Clinical Research Center for Laryngopharyngeal and Voice Disorders in Hunan Province, Xiangya Hospital, Central South University, Changsha, Hunan 410008 China; 5 Department of Hematology, The Third Xiangya Hospital, Central South University, Changsha, Hunan 410013 China; 6 Department of Dermatology, Hunan Key Laboratory of Medical Epigenomics, The Second Xiangya Hospital, Central South University, Changsha, Hunan 410011 China; 7 National Clinical Research Center for Geriatric Disorders, Xiangya Hospital, Central South University, Changsha, Hunan 410008 China; 8 Hospital for Skin Diseases (Institute of Dermatology), Chinese Academy of Medical Sciences and Peking Union Medical College, Nanjing, 210042 China; 9 Present Address: Department of Medical Imaging, Sun Yat-Sen University Cancer Center, Guangzhou, Guangdong 510060 China; 10 Present Address: Department of Dermatology, Hunan Children’s Hospital, 86 Ziyuan Road, Changsha, Hunan 410007 China; 11 Present Address: Department of Cancer Immunology and Virology, Dana-Farber Cancer Institute, Harvard Medical School, Boston, MA 02215 USA

**Keywords:** Immunization, Cytotoxic T cells, Transforming growth factor beta, Cancer microenvironment

## Abstract

TGF-β signaling is necessary for CD8^+^ T cell differentiation into tissue resident memory T cells (T_RM_). Although higher frequency of CD8^+^ T_RM_ cells in the tumor microenvironment is associated with better prognosis, TGF-β−blockade typically improves rather than worsens outcomes. Here we show that in a mouse melanoma model, in the tumor-draining lymph nodes (TDLN) rather than in the tumors themselves, stem-like CD8^+^ T cells differentiate into T_RM_s in a TGF-β and tumor antigen dependent manner. Following vaccination against a melanoma-specific epitope, most tumour-specific CD8^+^ T cells are maintained in a stem-like state, but a proportion of cells lost T_RM_ status and differentiate into CX3CR1^+^ effector CD8^+^ T cells in the TDLN, which are subsequently migrating into the tumours. Disruption of TGF-β signaling changes the dynamics of these developmental processes, with the net result of improving effector CD8^+^ T cell migration into the tumours. In summary, TDLN stem-like T cells transiently switch from a TGF-β-dependent T_RM_ differentiation program to an anti-tumor migratory effector development upon vaccination, which transition can be facilitated by targeted TGF-β blockade.

## Abstract (web summary)

TGF-β has been shown to regulate stem-like CD8 + T cell differentiation into tissue resident memory T cells in chronic infection. Here authors show that in tumour-bearing mice, a similar TGF-βdependent CD8 + T cell differentiation program is carried out in the draining lymph nodes, which impedes generation of anti-tumor migratory effector T cells upon future vaccination.

## Introduction

Persistent antigen exposure (e.g., tumor antigen) induces T cell exhaustion with reduced effector function^1^. Exhausted CD8^+^ T cells are heterogenous and a less exhausted subset carries stem cell-like features^2–6^. These stem-like CD8^+^ T cells express transcription factor Tcf-1 (T cell factor-1) and sustain CD8^+^ response during chronic antigen exposure. Importantly, these stem-like CD8^+^ T cells are the ones responding to immune checkpoint blockade therapies^3,5,7^ and correlating with the efficacy of tumor vaccines^8,9^. However, the signals that control the maintenance, differentiation, and migration of these stem-like T cells are not entirely known.

Transforming growth factor-β (TGF-β) is generally considered as an immune suppressor. Blocking TGF-β signaling has been demonstrated to boost tumor control via targeting tumor stromal compartment^10–13^ or CD4^+^ T cell-mediated blood vasculature remodeling^14,15^. In addition, systemic blocking TGF-β synergizes with tumor vaccine or PD-1/PD-L1 blockade to boost CD8^+^ T cell response in mouse models^16–20^. Because most studies about TGF-β on CD8^+^ T cells were carried out without incorporating the knowledge of stem-like T cells, it is critical to revisit the function of TGF-β on tumor-specific CD8^+^ T cells, especially on stem-like CD8^+^ T cells.

Tissue-resident memory T cells (T_RM_) represent a unique memory T cell population, which is separated from the circulation and maintained in a self-sustained manner^21–24^. Originally discovered in acute infection models, T_RM_ has been established as an essential component of tissue-specific immunity. Surprisingly, recent findings have demonstrated that stem-like CD8^+^ T cells generated after chronic viral infection bear similar properties as T_RM_, i.e., mostly confined to secondary lymphoid organs (e.g., spleen and lymph nodes) and non-circulating^25,26^. However, whether similar scenario exists in tumor immunity settings remains unknown. The vast majority of previous research on tumor-specific CD8^+^ T cells, including stem-like T cells is focused on tumor-infiltrating lymphocytes (TIL). Even though the cancer-immunity cycle model^27^ is widely accepted, tumor-specific CD8^+^ T cells residing in lymphoid organs is substantially underappreciated. It is well known that TGF-β signaling to CD8^+^ T cells is essential for the differentiation and maintenance of T_RM_ after acute infection^28–33^. When focusing on tumor immunity, T_RM_-like signature has often been positively associated with the capacity of TILs to control tumor^34–41^. It remains a mystery how to fully reconcile the facts that TGF-β promotes T_RM_, T_RM_ limits tumor growth and TGF-β blockade improves tumor control.

Here, we show that stem-like CD8^+^ T cells differentiate into T_RM_ inside tumor-draining lymph nodes (TDLN) in a TGF-β-dependent manner. Tumor vaccine induces transient loss of T_RM_ features, which is required for the differentiation of migratory effectors and superior anti-tumor responses. Our findings reveal a connection between T_RM_ and stem-like T cells inside TDLNs, which suppresses the migration and effector differentiation of stem-like T cells, leading to dampened anti-tumor immunity.

## Results

### Disruption of TGF-β receptor in tumor-specific CD8^+^ T cells alone is not sufficient for tumor control

To examine the role of TGF-β signaling in stem-like T cells during tumor immunotherapies, we generated mature T cell-specific TGF-β receptor conditional knockout mice (*Tgfbr2*^f/f^ distal *Lck*-Cre, hereafter referred to as *Tgfbr2*^*−/−*^)^42^. Distal *Lck*-Cre is only activated after thymocyte positive selection and has minimal impacts on thymocyte development^42^. Further, we established WT (wild type) and *Tgfbr2*^*−/−*^ Pmel-1 TCR transgenic mice carrying CD8^+^ T cells specific for an endogenous melanocyte epitope (gp100_25-33_ presented by H-2D^b^ ^43^). To be noted, gp100_25-33_ represents an endogenous tumor antigenic peptide derived from B16 melanoma. All our Pmel-1 mice (both WT and *Tgfbr2*^*−/−*^) carried congenic markers so that donor Pmel-1 T cells could be easily followed after adoptive transfer.

**Fig. 1:**
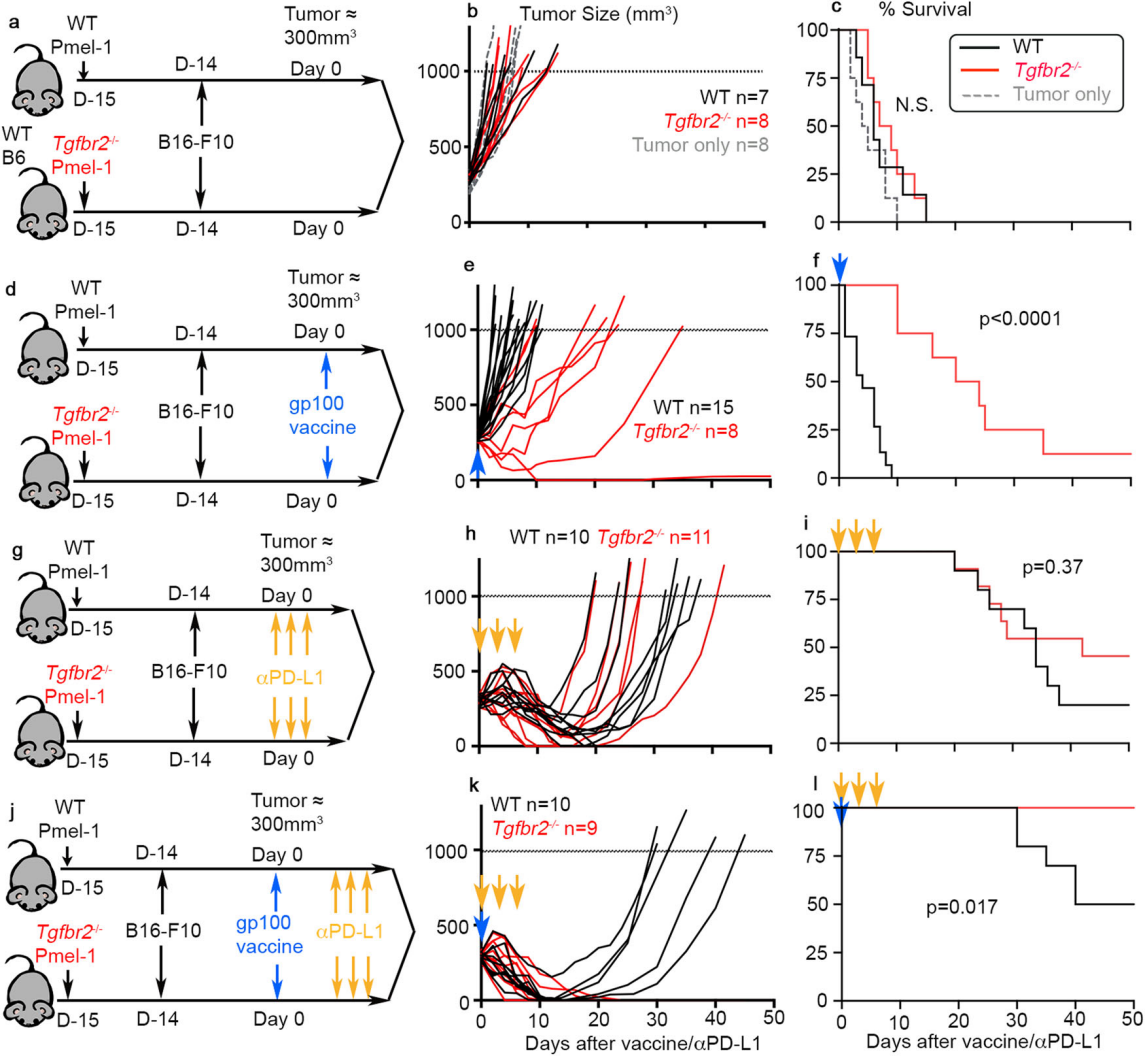
*Tgfbr2*^*−/−*^ CD8^+^ T cells exhibited greatly enhanced responses to tumor vaccine. **a**–**c** Pmel-1 alone. **a** Schematics; **b** tumor growth; **c** survival curve. No T cell transfer group is shown in gray dashed line. Black lines, WT Pmel-1 recipients and red lines, *Tgfbr2*^*−/−*^ recipients. For **b** and **c**, WT, *n* = 7, *Tgfbr2*^*−/−*^, *n* = 8 and no cell group, *n* = 8. **d**–**f** Pmel-1 +tumor vaccine (indicated as a blue arrow). **d** Schematics; **e** tumor growth; **f** Survival curve. For **e** and **f**, WT, *n* = 15, *Tgfbr2*^*−/−*^, *n* = 8. **g**–**i** Pmel-1 + αPD-L1 (indicated as yellow arrows). **g** Schematics; **h** tumor growth; **i** Survival curve. For **h** and **i**, WT, *n* = 10, *Tgfbr2*^*−/−*^, *n* = 11. **j**–**l** Pmel-1+tumor vaccine +  αPD-L1. **j** Schematics; **k** tumor growth; **l** Survival curve. For **k** and **l**, WT, *n* = 10, *Tgfbr2*^*−/−*^, *n* = 9. 2–3 independent repeats for each setting. Each line in **b**, **e**, **h** and **k** represents the results from an individual mouse. N.S., not significant (*p* > 0.05) and indicated *p* values were calculated by Mantel–Cox test. Two-sided tests were used. Source data are provided as a Source data file.

To mimic endogenous anti-tumor immunity, we adoptively transferred naive Pmel-1 T cells (10^5^ cells/mouse) into each unmanipulated WT mouse before tumor inoculation via a subcutaneous (s.c.) route. To be noted, donor Pmel-1 T cells function as a surrogate marker of endogenous tumor-specific CD8^+^ T cells. Distinct from therapeutic settings, freshly isolated naive Pmel-1 T cells were directly transferred without in vitro manipulation throughout our studies. To determine the efficacy of anti-tumor responses, naive WT and *Tgfbr2*^*−/−*^ Pmel-1 T cells were separately transferred into different recipients (Fig. 1a). Considering that only a small number of naive tumor-specific CD8^+^ T cells were transferred into a WT host with a full T cell compartment, no significant difference in tumor control was observed between WT mice which had vs had not received donor CD8 (Fig. 1b, c and S2b). Further, we did not observe significant difference in tumor control between recipients of WT vs *Tgfbr2*^*−/−*^ Pmel-1 T cells (Fig. 1b, c). We repeated the experiments with OT-1 TCR transgenic mice recognizing a peptide derived from chicken ovalbumin (OVA) and a B16-OVA tumor line. WT OT-1 and *Tgfbr2*^*−/−*^ OT-1 exhibited similar anti-tumor immunity in WT hosts (Fig. S2a and S2b). Our result suggests that suppression of TGF-β signaling in naturally primed CD8^+^ T cells alone is not sufficient to control aggressive tumors, such as B16 melanoma.

### *Tgfbr2*^*−/−*^ CD8^+^ T cells synergize with tumor vaccine but not with PD-L1 blockade

Next, we s.c. administrated a tumor vaccine [Poly I:C + gp100_25-33_ (Pmel-1 cognate peptide)] once when tumor size reached around 300 mm^3^ (Fig. 1d). Most likely due to the facts that tumor vaccine was given at a relatively late stage (around 300 mm^3^), anti-tumor immunity was not boosted in the recipients of WT Pmel-1 T cells (compare black lines in Fig. 1b vs 1e and Fig. 1c vs 1f). In contrast, *Tgfbr2*^*−/−*^ Pmel-1 cells elicited significantly improved tumor control after vaccination (Fig. 1e, f). Similar results were observed when B16-OVA and OT-1 system was used, i.e., *Tgfbr2*^*−/−*^ OT-1 exhibited significantly improved tumor control after administration of OVA peptide vaccine (Fig. S2c to S2e).

In contrast, PD-L1 blocking antibody boosted the anti-tumor immunity of both WT and *Tgfbr2*^*−/−*^ Pmel-1 T cells to a similar extent (compare Fig. 1b vs 1h and 1c vs 1i). In other words, there was no significant difference between WT and *Tgfbr2*^*−/−*^ Pmel-1 T cells after PD-L1 blockade (Fig. 1h, i). Finally, we combined αPD-L1 blocking antibody with tumor vaccine (Fig. 1j). Under this setting, WT Pmel-1+ vaccine + αPD-L1 exhibited significantly improved response with 50% of mice cured of tumor. In contrast, 100% of *Tgfbr2*^*−/−*^ Pmel-1+vaccine + αPD-L1 treated animals rapidly irradicated late-stage tumors (Fig. 1k, l).

Together, we have demonstrated that *Tgfbr2*^*−/−*^ CD8^+^ T cells exhibit superior response to tumor vaccine. Vaccine-activated CD8^+^ T cells further synergize with PD-L1 blockade therapy. Because the most striking difference between WT and *Tgfbr2*^*−/−*^ T cells was induced only after tumor vaccine, we would primarily focus on the response to tumor vaccine in the following studies.

### Greatly enhanced accumulation of *Tgfbr2*^*−/−*^ effector T cells in tumor after vaccination

**Fig. 2:**
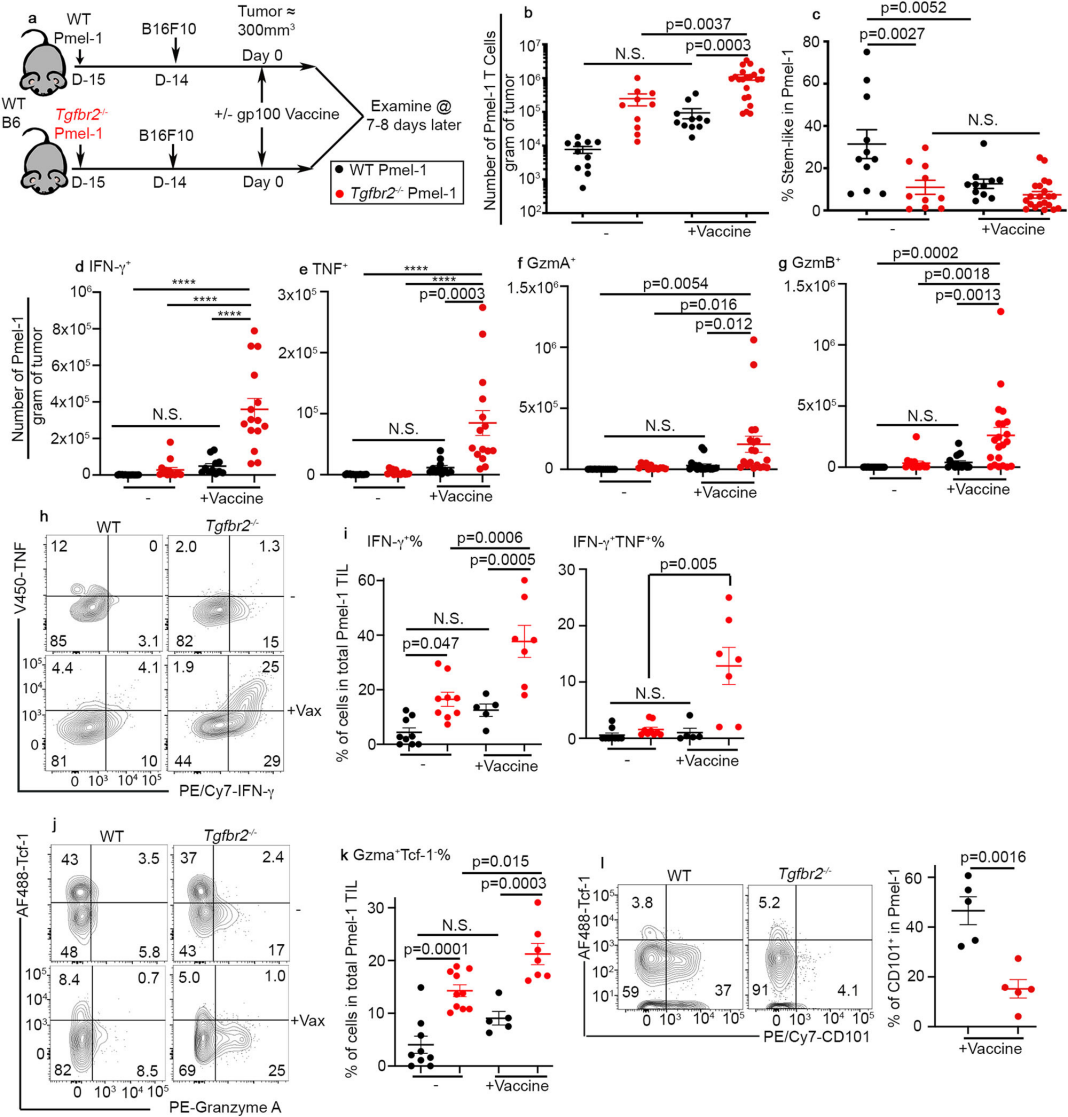
Increased accumulation of *Tgfbr2*^*−/−*^ effector CD8^+^ T cells inside tumor after vaccination. **a** Experimental design. Black symbols, WT and red symbols, *Tgfbr2*^*−/−*^. **b** The number of Pmel-1 T cells per gram of tumor is shown. **c** The percentage of stem-like subset in Pmel-1 T cells isolated from tumor is shown. For **b** and **c**, WT, *n* = 11, *Tgfbr2*^*−/−*^, *n* = 10, WT + Vaccine, *n* = 11 and *Tgfbr2*^*−/−*^+Vaccine, *n* = 21. Per gram of tumor, the numbers of Pmel-1 T cells producing IFN-γ (**d**), TNF (**e**), Granzyme A (**f**), and Granzyme B (**g**) are shown. For **d** and **e**, WT, *n* = 15, *Tgfbr2*^*−/−*^, *n* = 14, WT + Vaccine, *n* = 11 and *Tgfbr2*^*−/−*^+Vaccine, *n* = 15. For **f**, WT, *n* = 13, *Tgfbr2*^*−/−*^, *n* = 12, WT + Vaccine, *n* = 17 and *Tgfbr2*^*−/−*^+Vaccine, *n* = 19. For **g**, WT, *n* = 15, *Tgfbr2*^*−/−*^, *n* = 14, WT + Vaccine, *n* = 17 and *Tgfbr2*^*−/−*^+Vaccine, *n* = 21. **h** Representative FACS profiles of TIL Pmel-1 T cells are shown. **i** The percentage of IFN-γ^+^ (left) and IFN-γ^+^TNF^+^ TIL Pmel-1 T cells are shown. WT, *n* = 9, *Tgfbr2*^*−/−*^, *n* = 9, WT + Vaccine, *n* = 5 and *Tgfbr2*^*−/−*^+Vaccine, *n* = 7. Representative FACS (**j**) and the percentage of Granzyme A^+^Tcf-1^−^ effector T cells in TIL Pmel-1 (**k**) are shown. WT, *n* = 9, *Tgfbr2*^*−/−*^, *n* = 10, WT + Vaccine, *n* = 5 and *Tgfbr2*^*−/−*^+Vaccine, *n* = 7. **l** Left, representative FACS profiles of pre-gated donor Pmel-1 T cells isolated from tumor are shown; Right, the percentage of CD101^+^ subset in Pmel-1 T cells is shown. *N* = 5/each group. Each symbol represents the results from an individual mouse. 2–3 independent repeats. Data are presented as mean ± SEM. N.S., not significant (*p* > 0.05), ****p < 0.0001 and indicated *p* values are calculated by unpaired Student *t*-test (**l**) or Ordinary one-way ANOVA with multi-comparison posttest (**b**–**k**). Two-sided tests were used. Source data are provided as a Source data file.

To determine the mechanisms underlying improved response to tumor vaccine for *Tgfbr2*^*−/−*^ T cells, we first focused on TIL Pmel-1 T cells before and after vaccination (illustrated in Fig. 2a and gating strategies in Fig. S1). Indeed, tumor vaccine significantly boosted the total number of TIL *Tgfbr2*^*−/−*^ Pmel-1 T cells (Fig. 2b). In contrast, even though there was a similar trend, the difference of WT Pmel-1 T cells before vs after vaccination did not reach statistical significance (Fig. 2b). Interestingly, the stem-like subset of TIL Pmel-1 T cells was significantly reduced for *Tgfbr2*^*−/−*^ cells before vaccination. WT and *Tgfbr2*^*−/−*^ Pmel-1 T cells carried a similar stem-like subset after vaccination (Fig. 2c). When B16-OVA and OT-1 system was examined, *Tgfbr2*^*−/−*^ OT-1 T cells exhibited significantly enhanced accumulation inside tumor after vaccination (Fig. S2f). No significant difference of Tcf-1^+^ stem-like T cells was detected between WT vs T*gfbr2*^−/−^ OT-1 T cells before or after vaccination (Fig. S2g).

We further confirmed that only after vaccination, TIL *Tgfbr2*^*−/−*^ Pmel-1 T cells exhibited greatly enhanced effector functions, including the production of IFN-γ, TNF, granzyme A, and granzyme B (Fig. 2d, g). The enhanced effector function was confirmed both for total cell number (Fig. 2d, g) and the percentage of Pmel-1 T cells actively producing effector molecules (Fig. 2h-k). Consistently, TIL *Tgfbr2*^*−/−*^ Pmel-1 T cells (Fig. 2l) and *Tgfbr2*^*−/−*^ OT-1 T cells (Fig. S2h) carried significantly reduced population of terminally exhausted CD101^+^ subset after vaccination^44,45^. Together, enhanced tumor control (Fig. 1) is likely due to greatly enhanced accumulation of *Tgfbr2*^*−/−*^ effector T cells infiltrating the tumors after vaccination.

### Cell migration is required for *Tgfbr2*^*−/−*^ Pmel-1 T cells to respond to vaccination

**Fig. 3:**
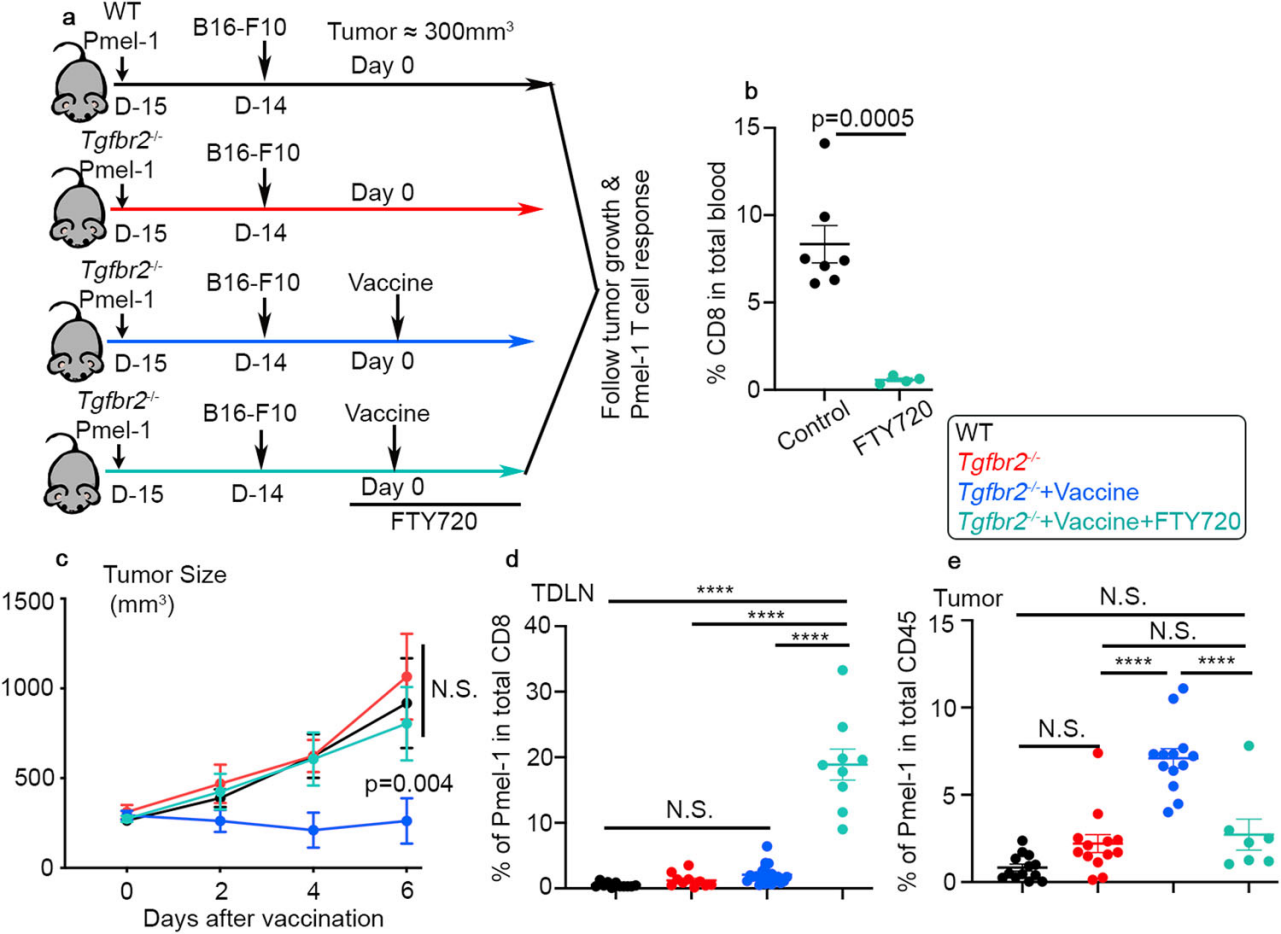
Cell migration is required for *Tgfbr2*^*−/−*^ Pmel-1 T cells to respond to tumor vaccine. **a** Experimental design. Black, WT-vaccine; red, *Tgfbr2*^−/−^-vaccine; blue, *Tgfbr2*^−/−^+vaccine; and aqua, *Tgfbr2*^−/−^+vaccine+FTY720. **b** The percentage of CD8^+^ T cells in red blood cell lysed total peripheral blood are shown (control, *n* = 7 and FTY720, *n* = 4). **c** Tumor size (*n* = 4 for WT group and *n* = 5 for *Tgfbr2*^*−/−*^ groups). Statistical analysis was performed on day 6 results. **d** The percentage of donor Pmel-1 T cells in total CD8^+^ T cells isolated from TDLN is shown (WT, *n* = 11, *Tgfbr2*^*−/−*^, *n* = 10, *Tgfbr2*^*−/−*^+Vaccine, *n* = 21, and *Tgfbr2*^*−/−*^+Vaccine+FTY720, *n* = 9). **e** The percentage of donor Pmel-1 T cells in total CD45^+^ cells isolated from tumor is shown (WT, *n* = 13, *Tgfbr2*^*−/−*^, *n* = 13, *Tgfbr2*^*−/−*^+Vaccine, *n* = 13, and *Tgfbr2*^*−/−*^+Vaccine+FTY720, *n* = 7). Each symbol in **b**, **d**, and **e** represents the results from an individual mouse. Data are presented as mean ± SEM. 3 independent repeats. N.S., not significant (*p* > 0.05), ****p < 0.0001 and indicated *p* values are calculated by Ordinary one-way ANOVA with multi-comparison posttest **c**–**e** or Student *t*-test **b**. Two-sided *t*ests were used. Source data are provided as a Source data file.

It has been shown that stem-like T cells are the ones responding to tumor vaccine^8,9^. We had been puzzled by our findings that *Tgfbr2*^*−/−*^ TILs carried less (Fig. 2c) or similar (Fig. S2g) stem-like subset before vaccination and exhibited enhanced response to tumor vaccine (Figs. 1, 2 and Fig. S2). Considering widely accepted cancer-immunity cycle^27^, we wondered whether active communication between lymphoid organs and tumors was involved. To this end, we employed FTY720, a small molecule targeting S1PR1 (Sphingosine-1-phosphate Receptor 1) and inhibiting T cell egress from lymphoid organs. We started FTY720 treatment one day before vaccination to ensure stable concentration of FTY720 reached inside experimental animals (illustrated in Fig. 3a). As expected, FTY720 treatment efficiently suppressed CD8 T cell circulation in the blood (Fig. 3b). Consistently, tumor vaccine elicited greatly enhanced tumor control in mice received *Tgfbr2*^*−/−*^ Pmel-1 T cells. FTY720 treatment completely abolished this response (Fig. 3c). Further, FTY720 significantly enhanced the population of *Tgfbr2*^*−/−*^ Pmel-1 T cells inside TDLNs (Fig. 3d) while abolished the increase of TIL *Tgfbr2*^*−/−*^ Pmel-1 T cells after vaccination (Fig. 3e). This result clearly demonstrates that cell migration from TDLN to tumor is essential for *Tgfbr2*^*−/−*^ Pmel-1 T cells to respond to tumor vaccine.

Further, it has been demonstrated that CX3CR1^+^ effector CD8^+^ T cells representing a migratory subset with enhanced effector functions^46^. Indeed, we found that CX3CR1^+^ Pmel-1 T cells were undetectable before vaccination in TDLN. Tumor vaccine greatly boosted CX3CR1^+^ subset in TDLN, especially for *Tgfbr2*^*−/−*^ cells (Fig. S3a and S3b), leading to significantly increased accumulation of this effector subset in the tumors (Fig. S3c). Together, tumor vaccine induces the differentiation of migratory effectors in TDLN and TGF-β suppresses this process.

### TDLN represents a unique tissue to host T_RM_ stem-like CD8^+^ T cells

It is well established that TGF-β provides an essential signal for T_RM_ differentiation after acute infections^28–33^. Stem-like T cells largely reside inside secondary lymphoid organs without circulation during chronic LCMV (lymphocytic choriomeningitis virus) infection^25^. Based on these facts and our findings that *Tgfbr2*^*−/−*^ Pmel-1 T cells exhibited enhanced migration from TDLN to tumor after vaccination, we hypothesized that stem-like Pmel-1 T cells differentiate into T_RM_ inside TDLN in a TGF-β-dependent manner.

**Fig. 4:**
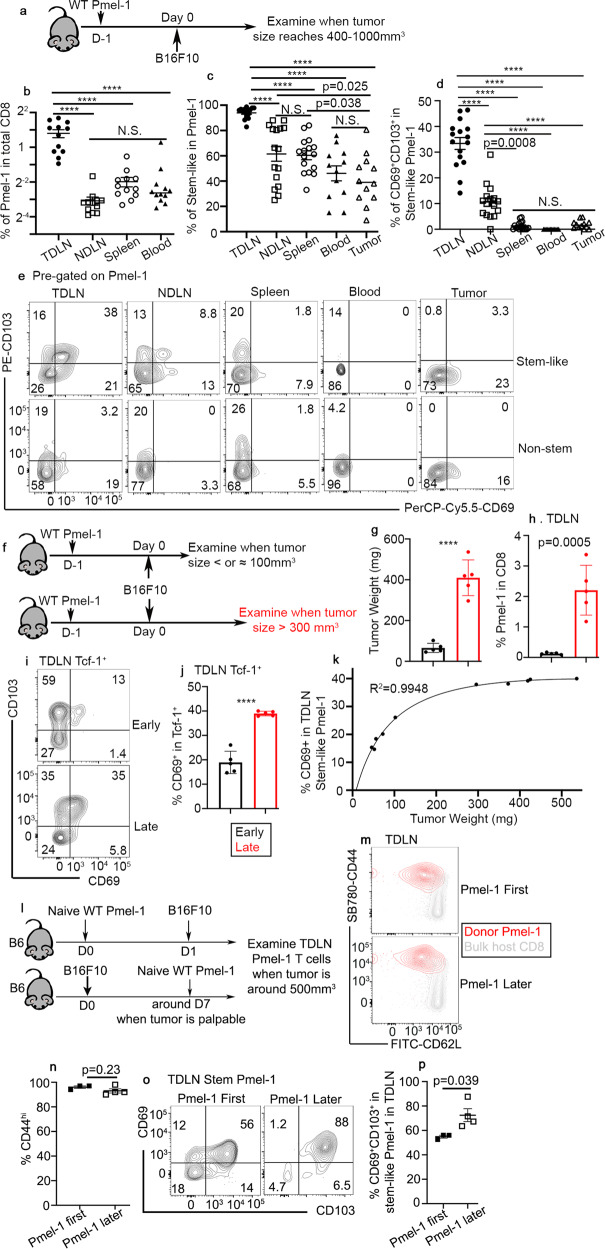
Late-stage tumors facilitate the differentiation of T_RM_ stem-like T cells in TDLN. **a** Experimental design for (**b**) to (**e**). Filled circle, TDLN, empty square, NDLN, empty circle, spleen, filled triangle, blood and empty triangle, tumor. The percentage of Pmel-1 T cells in total CD8^+^ cells (**b**, *n* = 12/each), the percentage of stem-like subset in Pmel-1 T cells (**c**) and the percentage of CD69^+^CD103^+^ cells in stem-like Pmel-1 T cells (**d**) isolated from different tissues of tumor-bearing mice are shown. For **c** and **d**, *n* = 16 for TDLN, NDLN, and spleen; *n* = 12 for blood and tumor. **e** Representative FACS profiles of stem-like (Upper) and non-stem (Lower) Pmel-1 T cells isolated from different tissues are shown. **f** Experimental design for (**g**) to (**k**). Black, early tumor and red, later tumor. **g** Tumor weight for early vs late time points is shown (*n* = 5/each). **h** The percentage of Pmel-1 T cells in total CD8 in TDLN is shown (*n* = 5/each). **i** Representative FACS profiles of pre-gated Tcf-1^+^ Pmel-1 T cells from TDLN are shown. **j** The percentage of CD69^+^ subset in Tcf-1^+^Pmel-1 T cells isolated from TDLN is shown (*n* = 5/each). **k** Nonlinear regression of the percentage of CD69^+^ among Tcf-1^+^Pmel-1 T cells in TDLN vs tumor weight of the same animal (*n* = 10). **l** Experimental design for (**m**) to (**p**). Filled square, Pmel-1 first and empty square, Pmel-1 later. **m** Representative FACS profiles of TDLN Pmel-1 T cells overlaid with host CD8^+^ T cells are shown. **n** The percentage of CD44^hi^ cells in TDLN Pmel-1 T cells are shown (Pmel first, *n* = 3 and Pmel later, *n* = 4). **o** Representative FACS profiles of pre-gated Tcf-1^+^ Pmel-1 T cells in TDLN are shown. **p** The percentage of CD69^+^CD103^+^ cells in Tcf-1^+^Pmel-1 T cells are shown (Pmel first, *n* = 3 and Pmel later, *n* = 4). Data are presented as mean ± SEM. Each symbol represents the results from an individual recipient. N.S., not significant (*p* > 0.05), ****p < 0.0001 and indicated *p* values are calculated by Ordinary one-way ANOVA with multi-comparison posttest (**b**–**d**) or Student *t*-test (**g**–**p**). Two-sided tests were used. Source data are provided as a Source data file.

To test this hypothesis, we employed widely accepted T_RM_ marker CD69 and CD103, and first focused on WT Pmel-1 T cells. As shown in Fig. 4a, naive WT Pmel-1 T cells were adoptively transferred into B6 hosts followed by B16F10 tumor inoculation. After tumor grew to more than 400 mm^3^, the distribution and phenotype of donor Pmel-1 T cells were examined. As expected, tumor-specific Pmel-1 T cells were highly enriched in TDLNs (Fig. 4b). Interestingly, stem-like T cells were also enriched in TDLNs (Fig. 4c). Other secondary lymphoid organs [including both non-draining LN (NDLN) and spleen] harbored less stem-like T cells than TDLNs, but more than tumors (Fig. 4c). Importantly, CD69^+^CD103^+^ T_RM_ phenotype was largely restricted to stem-like T cells isolated from TDLNs (Fig. 4d, e).

Next, we examined the kinetics of stem-like T cells to adopt a T_RM_ phenotype. To this end, after adoptive WT Pmel-1 transfer and B16F10 inoculation, we examined the mice either at an early time point when tumor size was less than 100 mm^3^ or at a late time point when tumor size reaches around 400 mm^3^ (illustrated in Fig. 4f, tumor weight in Fig. 4g). As expected, significantly increased Pmel-1 T cells were detected in large tumor TDLNs (Fig. 4h). Importantly, T_RM_ phenotype, especially the induction of CD69 on stem-like T cells was enhanced in late stage TDLNs (Fig. 4i, j). The percentage of CD69 on TDLN stem-like T cells was correlated very well with tumor size at early stage. After tumor weight reached around 300 mg, the percentage of T_RM_ subset in TDLN stem-like T cells was largely plateaued (Fig. 4k).

In addition, we performed a side-by-side comparison between different Pmel-1 transfer time (Fig. 4l). In this setting, naive Pmel-1 T cells were either primed at an early stage when tumor was inoculated OR at a late stage when tumor was already established. Both early and late tumor efficiently primed naive Pmel-1 T cells (Fig. 4m, n). Interestingly, late-stage tumor induced enhanced CD69^+^CD103^+^ stem-like Pmel-1 T cell differentiation in TDLNs (Fig. 4o, p).

To further validate this finding, we extended our analysis to bulk endogenous polyclonal CD8^+^ T cells. Indeed, we could consistently detect a small subset of Slamf6^+^Tcf-1^+^ endogenous CD8^+^ T cells, presumably representing stem-like CD8^+^ T cells (Fig. S4a). A substantial portion of these stem-like T cells, but not other endogenous CD8^+^ subsets, expressed both CD103 and CD69 (Fig. S4a). To be noted, Tcf-1^+^Slamf6^-^ subset likely included non-specific naive CD8^+^ T cells that expressed CD103, but not CD69. Importantly, identical to donor Pmel-1 T cells (Fig. 4d, e), when comparing stem-like endogenous CD8^+^ T cells isolated from different lymphoid organs, there was a stepwise reduction of T_RM_ phenotype from TDLN, NDLN to spleen (Fig. S4b).

Together, we have demonstrated that the vast majority of tumor-specific Pmel-1 T cells maintain as stem-like in TDLNs. A significant portion of stem-like CD8^+^ T cells in TDLNs acquire a T_RM_ phenotype, which is facilitated by the microenvironment of established tumor. Importantly, this finding is not limited to monoclonal Pmel-1 T cells and can be validated in polyclonal CD8^+^ population.

### CD4 help, TGF-β, and tumor antigen together induce T_RM_ phenotype on stem-like T cells in TDLN

**Fig. 5:**
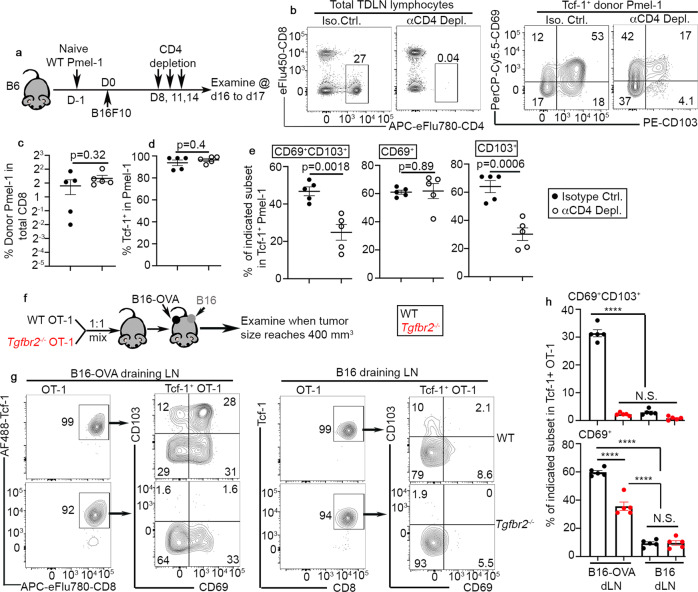
CD4 help, tumor antigen, and TGF-β-dependent establishment of TRM stem-like T cells in TDLN. **a** Experimental design for (**b**) to (**e**). Filled circle, control and empty circle, CD4 depleted. **b** Representative FACS profiles of total TDLN lymphocytes (left) and pre-gated Tcf-1^+^Pmel-1 T cells (right) are shown. **c** The percentage of Pmel-1 T cells in total TDLN CD8^+^ T cells are shown. **d** The percentage of Tcf-1^+^ cells in TDLN Pmel-1 T cells are shown. **e** The percentage of CD69^+^CD103^+^ (left), CD69^+^ (middle), and CD103^+^ (right) in Tcf-1^+^ Pmel-1 T cells are shown. For (**c**) to (**e**), *n* = 5/each. **f** Experimental design for (**g**) and **h**. Black, WT and red, *Tgfbr2*^*−/−*^. **g** Representative FACS profiles of donor OT-1 T cells isolated from B16-OVA draining LN (Left) and B16 draining LN (Right) are shown. **h** The percentage of CD69^+^CD103^+^ cells in stem-like OT-1 T cells (Upper) and the percentage of CD69^+^ cells in stem-like OT-1 T cells (Lower) are shown (*n* = 5/each). Data are presented as mean ± SEM. Each symbol represents the results from an individual recipient. N.S., not significant (*p* > 0.05), ****p < 0.0001 and indicated *p* values are calculated by Student *t*-test (**c**–**e**) and Ordinary one-way ANOVA with multi-comparison posttest (**h**). Two-sided tests were used. Source data are provided as a Source data file.

To dissect the cellular interactions underlying T_RM_-stem-like T cell differentiation, we tested the role of CD4^+^ T cells. Briefly, we employed a transient depletion system after initial phase of CD8^+^ T cell response and tumor establishment (Fig. 5a). As shown in Fig. 5b, CD4^+^ T cells were efficiently depleted. Total Pmel-1 T cell expansion (Fig. 5c) and total stem-like CD8^+^ T cells (i.e., Tcf-1^+^, Fig. 5d) were not impacted by CD4 depletion. In contrast, CD69^+^CD103^+^ T_RM_ stem-like cells were significantly reduced in the absence of CD4^+^ T cells (Fig. 5b right and 5e left). Interestingly, the reduction of CD69^+^CD103^+^ cells was largely due to the loss of CD103 expression (Fig. 5e right) while the expression of CD69 was not affected (Fig. 5e middle). Thus, CD4 help is required for efficient differentiation of T_RM_ stem-like CD8^+^ T cells in TDLNs.

To address the question why T_RM_-stem-like T cells were enriched in TDLNs, we examined the impacts of tumor antigen. To this end, we employed two B16 tumor lines, one with constitutive expression of model antigen OVA (B16-OVA) and one without (B16). As illustrated in Fig. 5f, WT and *Tgfbr2*^*−/−*^ OT-1 were adoptively co-transferred into B6 hosts. All host mice were s.c. inoculated with B16 and B16-OVA on the opposite sides. In this system, the contribution from TGF-β and tumor antigen could be precisely determined in the same animal. As shown in Fig. 5g, h, WT Tcf-1^+^ OT-1 differentiated into CD69^+^CD103^+^ cells in B16-OVA-draining LN, but not in B16-draining LN. The expression of CD103 was TGF-β-dependent. Consistent with the findings in acute infection-induced T_RM_^33,47,48^, the induction of CD69 was reduced, but not completely abolished in *Tgfbr2*^*−/−*^ cells. Importantly, considering the active trafficking from one LN to the next for most migratory T cells, the highly restricted distribution of CD69^+^CD103^+^ OT-1 in B16-OVA-draining LN, but not in B16-draining LN demonstrated that CD69^+^CD103^+^ cells were bona fide T_RM_ cells without circulation.

Together, we have demonstrated that naturally primed tumor-specific CD8^+^ T cells differentiate into T_RM_s in TDLNs in a CD4 help-, TGF-β-, and tumor antigen-dependent manner.

### T_RM_ stem-like CD8^+^ T cells in TDLNs are antigen-experienced T cells

**Fig. 6:**
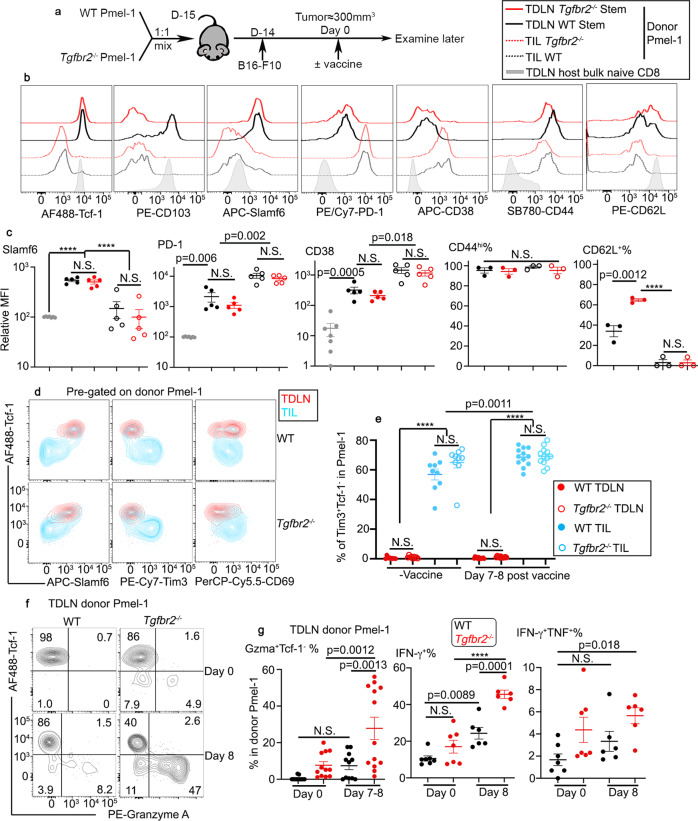
Tcf-1^+^ Pmel-1 T cells in TDLN are antigen-experienced T cells and differentiate into Tcf-1^-^ effectors in response to tumor vaccine. **a** Experimental design. **b**–**d** In the absence of tumor vaccine. **e**–**g** Side-by-side comparison of unvaccinated vs d7–8 post vaccine. **b** Representative histograms of pre-gated Tcf-1^+^ Pmel-1 T cells isolated from TDLN, total TIL Pmel-1 T cells and host-derived bulk naive CD8 are shown. In **b**, black line, WT and red line, *Tgfbr2*^−/−^; solid line, TDLN Tcf-1^+^Pmel-1; dotted line, TIL Pmel-1; and gray shade, bulk TDLN CD8. **c** Normalized MFI of Slamf6, PD-1 and CD38 as well as the percentage of CD44^hi^ and CD62L^+^ cells in Tcf-1^+^ TDLN Pmel-1 T cells and TIL Pmel-1 T cells are shown. For Slamf6, PD-1 and CD38, bulk LN CD8, *n* = 7 and other groups, *n* = 5. For bulk endogenous CD8 and LN stem-like Pmel-1 T cell comparison, tumor samples were excluded. For CD44 and CD62L, *n* = 3/each. In **c**, gray filled circle, bulk LN CD8; black, WT; red, *Tgfbr2*^*−/−*^; filled, TDLN Tcf-1^+^ Pmel-1; and empty, TIL Pmel-1. **d** Representative FACS profiles of TDLN Pmel-1 T cells overlaid with TIL Pmel-1 T cells isolated from the same animal. **e** The percentage of Tim3^+^Tcf-1^−^ cells in Pmel-1 T cells are shown (No Vaccine groups, *n* = 9/each and Vaccine groups, *n* = 12/each). In **d** and **e**, red, TDLN; blue, TIL; filled, WT; and empty, *Tgfbr2*^−/−^. **f** Representative FACS profiles of TDLN Pmel-1 T cells are shown. **g** The percentage of Granzyme A^+^Tcf-1^−^ (left, no Vaccine groups, *n* = 12, WT + Vaccine, *n* = 11, and *Tgfbr2*^*−/−*^+Vaccine, *n* = 13), IFN-γ^+^ (middle), and IFN-γ^+^TNF^+^ (right) in TDLN Pmel-1 T cells are shown (No Vaccine groups, *n* = 7/each and Vaccine groups, *n* = 6/each). Black, WT and red, *Tgfbr2*^*−/−*^. Each symbol in **c**, **e**, and (**g**) represents the results from an individual mouse. 2–3 independent repeats. Data are presented as mean ± SEM. N.S., not significant (*p* > 0.05), ****p < 0.0001 and indicated *p* values are calculated by Ordinary one-way ANOVA with Tukey’s multi-comparison posttest. Two-sided tests were used. Source data are provided as a Source data file.

We have briefly confirmed that WT Pmel-1 T cells are antigen-experienced in TDLNs (Fig. 4m, n). Because both CD103 and Tcf-1 are expressed in mouse naive CD8^+^ T cells (Fig. 6b), we would like to definitively rule out the possibility that Pmel-1 T cells were contaminated by naive cells in our system. To this end, we employed WT and *Tgfbr2*^*−/−*^ Pmel-1 co-transfer system (Fig. 6a). As shown in Fig. 6b, TDLN stem-like Pmel-1 T cells express CD103 (similar to naive CD8^+^ T cells) in a TGF-β-dependent manner. Distinct from naive counterparts, both WT and *Tgfbr2*^*−/−*^ stem-like Pmel-1 T cells carried higher levels of Slamf6, PD-1, CD38, and CD44, and lower levels of CD62L (Fig. 6b, c). We did observe higher levels of CD62L expression in *Tgfbr2*^*−/−*^ cells than WT controls (Fig. 6b, c), which may be explained by defective acquisition of T_RM_ program in the absence of TGF-β signaling. Further, considering that only a small number of naive Pmel-1 T cells were adoptively transferred, without extensive proliferation, it would be impossible to clearly detect donor T cells in various organs. Together, we conclude that T_RM_ stem-like Pmel-1 T cells are antigen-experienced.

### Dynamic regulation of stem-like T cells in TDLNs after vaccination

**Fig. 7:**
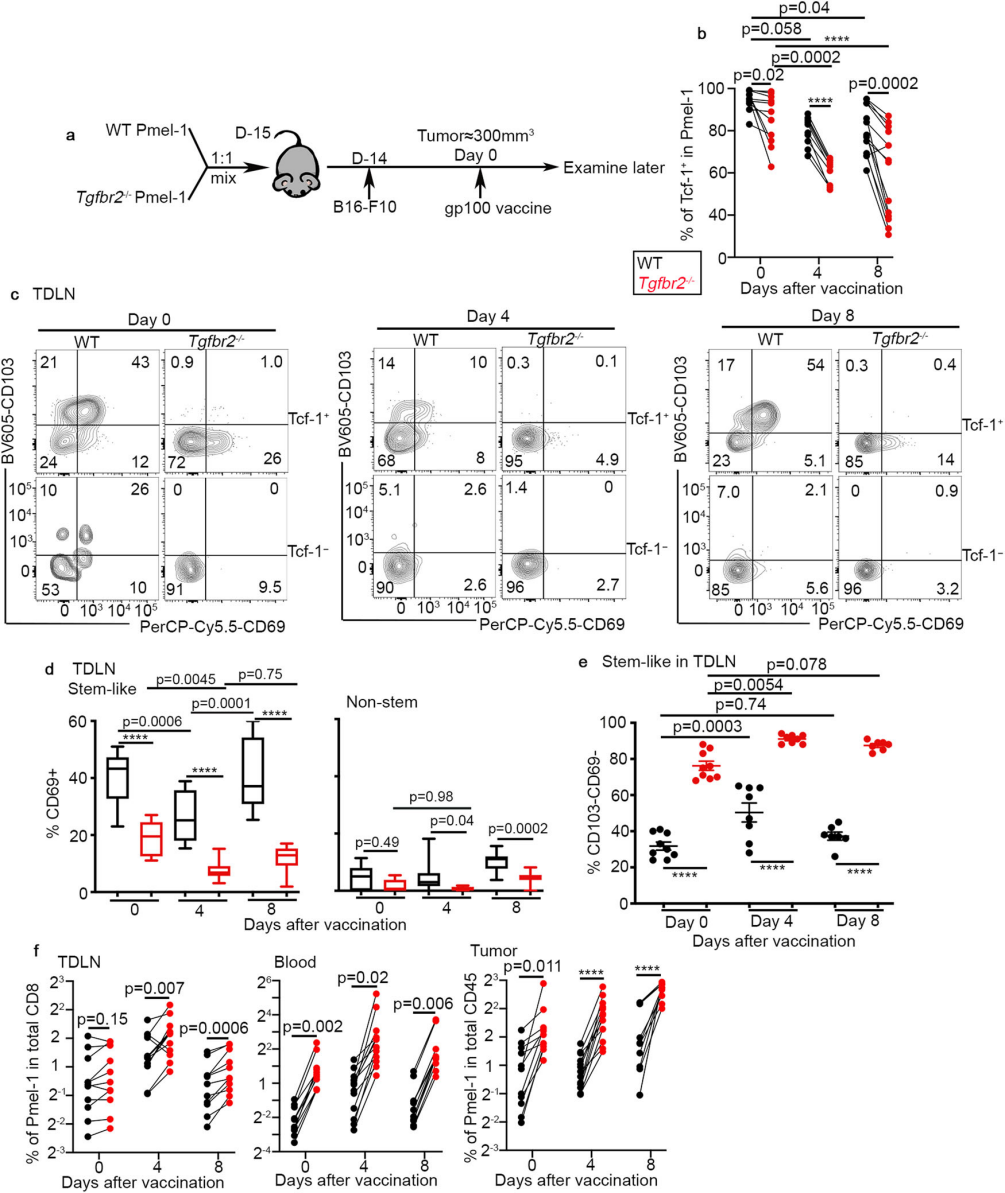
Tumor vaccine induces the loss of tissue residency in TDLN for stem-like T cells, accompanied by further differentiation into migratory effectors. **a** Experimental design. Black, WT and red, *Tgfbr2*^*−/−*^. **b** The percentage of Tcf-1^+^ stem-like subset in TDLN Pmel-1 T cells at different time points post vaccination are shown (d0, *n* = 12/each, d4, *n* = 10/each and d8, *n* = 14/each). **c** Representative FACS profiles of pre-gated Tcf-1^+^ (Upper, stem-like) and Tcf-1^-^ (Lower, non-stem) Pmel-1 T cells in TDLN at different time points after vaccination are shown. **d** The percentage of CD69^+^ cells in stem-like (Left) and non-stem (Right) Pmel-1 T cells isolated from TDLN are shown (For stem-like, d0 and d4, *n* = 13/each, d8, WT, *n* = 11 and *Tgfbr2*^*−/−*^, *n* = 13; for non-stem, d0, *n* = 9/each, d4, *n* = 13/each, d8, WT, *n* = 9 and *Tgfbr2*^*−/−*^, *n* = 13). **e** The percentage of non-T_RM_ (CD69^-^CD103^-^) cells in stem-like Pmel-1 T cells isolated from TDLN is shown (d0, *n* = 9/each, d4, *n* = 8/each and d8, *n* = 7/each). **f** The percentage of donor Pmel-1 T cells in total CD8^+^ (Left, TDLN; Middle, blood) or total CD45^+^ cells (Right, tumor) are shown (d0, *n* = 11/each, d4, *n* = 13/each and d8, *n* = 11/each). Each pair of symbols in **b** and **f**, each symbol in **e** represents the results from an individual recipient mouse. 3–4 independent repeats. Data are presented as mean ± SEM. Indicated *p* values and ****p < 0.0001 are calculated by Ordinary one-way ANOVA with multi-comparison posttest (unpaired samples) or paired Student *t*-test (paired samples from the same time point in **b** and **f**). Two-sided tests were used. Source data are provided as a Source data file.

Next, we focused on the response of TDLN stem-like T cells to tumor vaccine. To perform side-by-side comparison of WT vs *Tgfbr2*^*−/−*^ T cells isolated from the same tissue of the same animal, we employed adoptive co-transfer system. As illustrated in Fig. 7a, naive Pmel-1 T cells were isolated from congenically distinct WT and *Tgfbr2*^*−/−*^ mice, mixed at a 1:1 ratio and adoptively co-transferred into C57BL/6 recipients followed by s.c. B16 tumor inoculation. Tumor vaccine was administrated similarly at a late stage. The vast majority of TDLN WT Pmel-1 T cells exhibited a stem-like phenotype before vaccination (Fig. 7b day 0). Lack of TGF-β signaling moderately reduced the portion of stem-like T cells at base line (Fig. 7b day 0). After vaccination, stem-like T cells were slightly decreased for WT Pmel-1 T cells. In contrast, *Tgfbr2*^*−/−*^ Pmel-1 T cells exhibited a much more significant reduction of stem-like subset after vaccination (Fig. 7b), consistent with enhanced differentiation of stem→migratory effectors in *Tgfbr2*^*−/−*^ cells.

Importantly, this dynamic regulation of stem-like subset in response to tumor vaccine is TDLN-specific. In NDLNs, we did not detect any significant changes in the percentage of stem-like subset (Fig. S5a). In the spleen, we did find a reduction of stem-like subset in *Tgfbr2*^*−/−*^ T cells compared with WT counterparts. However, we did not observe tumor vaccine-induced alterations (Fig. S5b).

Interestingly, we found that in TDLNs, either before or after vaccination, we could not detect significant number of Tim-3^+^Tcf-1^-^ terminally exhausted T cells (Fig. 6d, e), further strengthening our findings that TDLN represents a unique tissue to host stem-like T cells. In response to vaccination, we observed significantly increased effector CD8 T cells producing IFN-γ, TNF and granzyme A, especially for *Tgfbr2*^*−/−*^ cells in TDLNs (Fig. 6f, g). We further validated this finding in TDLNs isolated from B16-OVA/OT-1 system (Fig. S2i and S2j).

Together, we find that in response to tumor vaccine, TDLN stem-like T cells differentiate into non-stem effectors and TGF-β inhibits this differentiation process.

### Tumor vaccine induces transient loss of T_RM_ phenotype in TDLN stem-like T cells

Then, we focused on the impacts of tumor vaccine on the T_RM_ phenotype of stem-like T cells in TDLNs. Consistent with Fig. 4 to Fig. 6, we found that before vaccine administration, a significant portion of stem-like T cells exhibited a T_RM_ phenotype for WT Pmel-1 T cells while the expression of CD103 was completely abolished and that of CD69 was significantly reduced in *Tgfbr2*^*−/−*^ ones (Fig. 7c left panel/top row, and 7d left). Shortly after vaccination (i.e., day 4), WT stem-like Pmel-1 T cells carried greatly reduced T_RM_ markers while *Tgfbr2*^*−/−*^ stem-like ones almost completely lost CD69 expression (Fig. 7c left vs middle, top row and 7d left). Day 8 after vaccination, WT stem-like T cells largely regained T_RM_ markers while *Tgfbr2*^*−/−*^ ones still carried reduced level of CD69 (Fig. 7c left vs right, top row and 7d left). If we could define CD69^−^CD103^−^ Tcf-1^+^ cells as non-T_RM_ stem-like cells, this subset was transiently induced in WT Pmel-1 T cells by tumor vaccine (Fig. 7e). In contrast, *Tgfbr2*^−/−^ Pmel-1 T cells carried significantly increased population of non-T_RM_ stem-like T cells before vaccination. Importantly, tumor vaccine further boosted sustained elevation of non-T_RM_ stem-like subset for *Tgfbr2*^*−/−*^ cells (Fig. 7e). Tcf-1^−^ effectors largely exhibited a non-T_RM_ migratory phenotype in TDLNs, especially after vaccination for both WT and *Tgfbr2*^*−/−*^ cells (Fig. 7c bottom row and 7d right).

The greatly reduced T_RM_ phenotype in TDLN *Tgfbr2*^*−/−*^ cells was translated into altered distribution. *Tgfbr2*^*−/−*^ Pmel-1 T cells only exhibited a subtle increase in TDLNs compared with co-transferred WT counterparts in the presence of vaccination (Fig. 7f left). In stark contrast, *Tgfbr2*^*−/−*^ Pmel-1 T cells were the dominant population detected in the blood (At day 0, 12 ± 4.4% WT vs 88 ± 4.4% *Tgfbr2*^−/−^; day 4, 14 ± 10% WT vs 86 ± 10% *Tgfbr2*^*−/−*^; day 8, 11 ± 2.4% WT vs 89 ± 2.4% *Tgfbr2*^*−/−*^ and Fig. 7f middle). The significantly increased circulation and migration of *Tgfbr2*^*−/−*^ Pmel-1 T cells led to markedly increased accumulation inside tumor, especially after vaccination (Fig. 7f right).

Interestingly, we could also detect similar tumor vaccine-induced changes of T_RM_ phenotype in Pmel-1 T cells isolated from NDLNs (Fig. S6a). However, we could not detect similar changes in either spleens (Fig. S6b) or tumors (Fig. S6c).

To rule out the possibility that our observation is B16 tumor-specific, we employed a different tumor model, i.e., colorectal cancer line MC38 expressing model antigenic peptide GP_33-41_ (MC38-GP_33-41_) together with P14 TCR transgenic mice carrying CD8^+^ T cells recognizing GP_33-41_ presented by MHC-I molecule H-2D^b^ (Fig. S7a). In a similar trend, TDLN represents a unique tissue to host Tcf-1^+^ P14 T cells (Fig. S7b). Different from B16 model, TIL CD8^+^ T cells expressed higher levels of CD69 in MC38 model (comparing Fig. S7c with Fig. 4e). However, in all secondary lymphoid organs, TDLN stem-like P14 T cells carried the highest levels of T_RM_ marker CD69 (Fig. S7c). Importantly, the expression of both CD69 and CD103 were similarly dependent on TGF-β signaling (Fig. S7f). Tumor vaccine induced WT stem-like P14 T cells to downregulate T_RM_ markers in TDLNs (Fig. S7g and h).

Together, tumor vaccine induces transient loss of T_RM_ phenotype in stem-like T cells in TDLNs, which coincides with the differentiation from stem-like T cells into migratory effectors. *Tgfbr2*^*−/−*^ CD8^+^ T cells carried significantly reduced T_RM_ phenotype at base line, and exhibited enhanced and prolonged response to tumor vaccine, i.e., increased differentiation into migratory effectors.

### Type I IFN-dependent adjuvant effects are required for vaccine-induced downregulation of T_RM_ phenotype

**Fig. 8:**
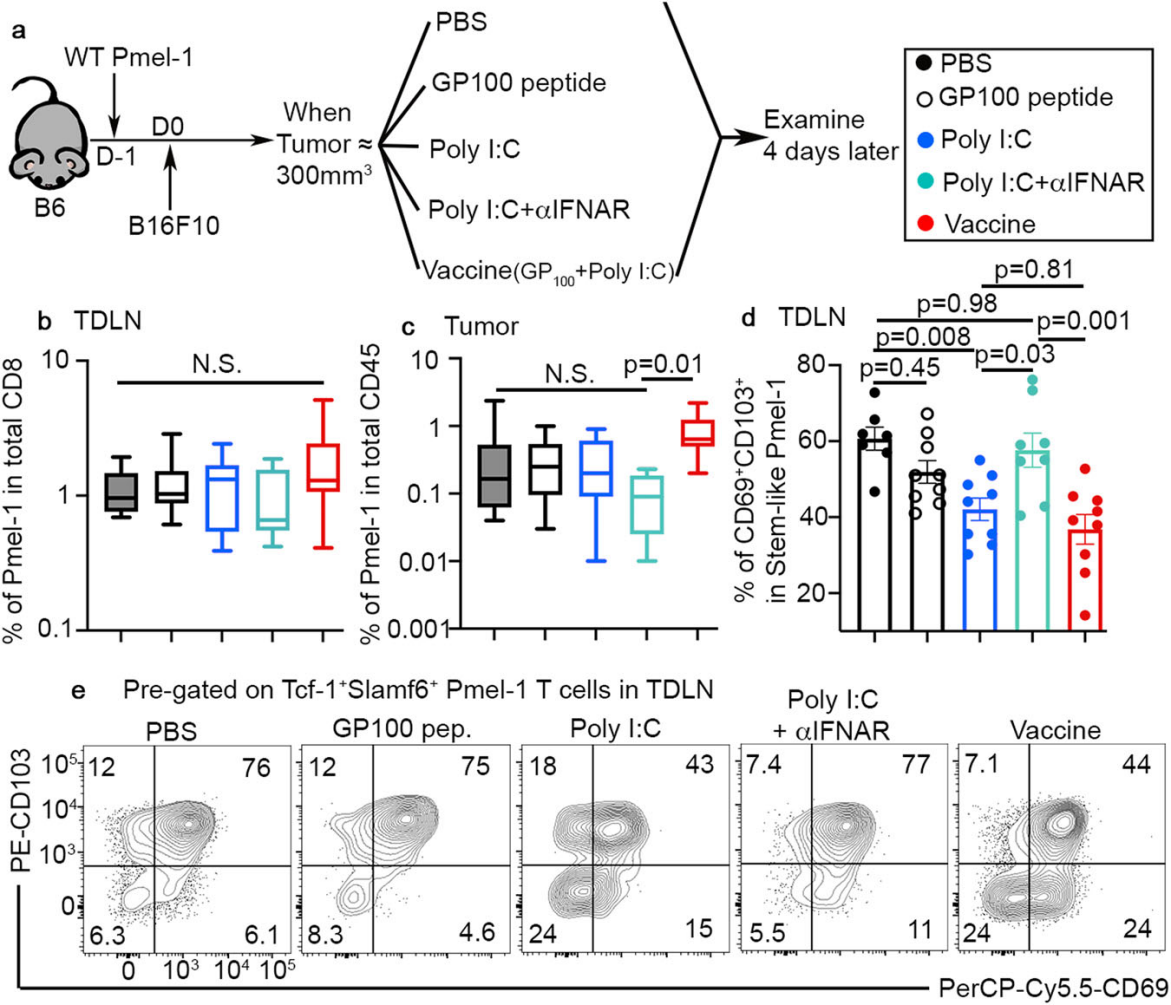
Loss of TRM phenotype in TDLN stem-like T cells is largely due to type I IFN-dependent adjuvant effects. **a** Experimental design. Filled black, PBS; empty black, peptide; blue, poly I:C; aqua, poly I:C + aIFNAR; and red, vaccine. **b** The percentage of donor Pmel-1 T cells in total TDLN CD8 are shown (PBS, *n* = 7, peptide, *n* = 9, poly I:C, *n* = 8, IFNAR blocking, *n* = 7 and Vaccine, *n* = 18). **c** The percentage of donor Pmel-1 T cells in total CD45^+^ cells isolated from the tumor are shown (PBS, *n* = 24, peptide, *n* = 9, poly I:C, *n* = 9, IFNAR blocking, *n* = 9 and Vaccine, *n* = 13). **d** The percentage of CD69^+^CD103^+^ cells in stem-like Pmel-1 T cells isolated from TDLN are shown (PBS, *n* = 7, peptide, *n* = 9, poly I:C, *n* = 9, IFNAR blocking, *n* = 8 and Vaccine, *n* = 9). **e** Representative FACS profiles of pre-gated stem-like Pmel-1 T cells isolated from TDLN are shown. Each symbol in **d** represents the results from an individual mouse. Data are presented as mean ± SEM. 2 independent repeats. N.S., not significant (*p* > 0.05) and indicated *p* values are calculated by Ordinary one-way ANOVA with multi-comparison posttest. Two-sided tests were used. Source data are provided as a Source data file.

We have demonstrated that tumor antigen is required for the establishment of T_RM_ features for stem-like T cells (Fig. 5). However, we have also found that tumor vaccine (which contains tumor antigenic peptide) induces the loss of T_RM_ markers (Fig. 7). To provide an explanation for this obvious paradox, we focused on the other tumor vaccine component, i.e., the adjuvant poly I:C. For this purpose, we employed a system illustrated in Fig. 8a. Briefly, naive WT Pmel-1 T cells were adoptively transferred into B6 recipients followed by tumor inoculation. About 2 weeks later, we ramdomly allocated the recipient mice into 5 groups, including PBS control, vaccine control, peptide alone, poly I:C alone, and poly I:C plus blocking antibody targeting type I IFN receptor. We focused our analysis on day 4 post treatment, when the loss of T_RM_ phenotypes reached its peak for WT cells (Fig. 7). Except for a slight increase of TIL Pmel-1 T cells for vaccine group, we did not detect significant changes in Pmel-1 T cells in both TDLNs and tumors across different groups (Fig. 8b, c). Remarkably, poly I:C alone induced significant reduction of CD69^+^CD103^+^ stem-like Pmel-1 T cells in TDLNs, which was indistinguishable from the results for vaccine group (Fig. 8d, e). Further, the effects of poly I:C were largely dependent on type I IFN (Fig. 8d, e). Together, we have demonstrated that adjuvant-induced type I IFN is essential for the loss of T_RM_ program in TDLN stem-like CD8^+^ T cells.

### WT Pmel-1 T cells are highly enriched for T_RM_ gene signature

**Fig. 9:**
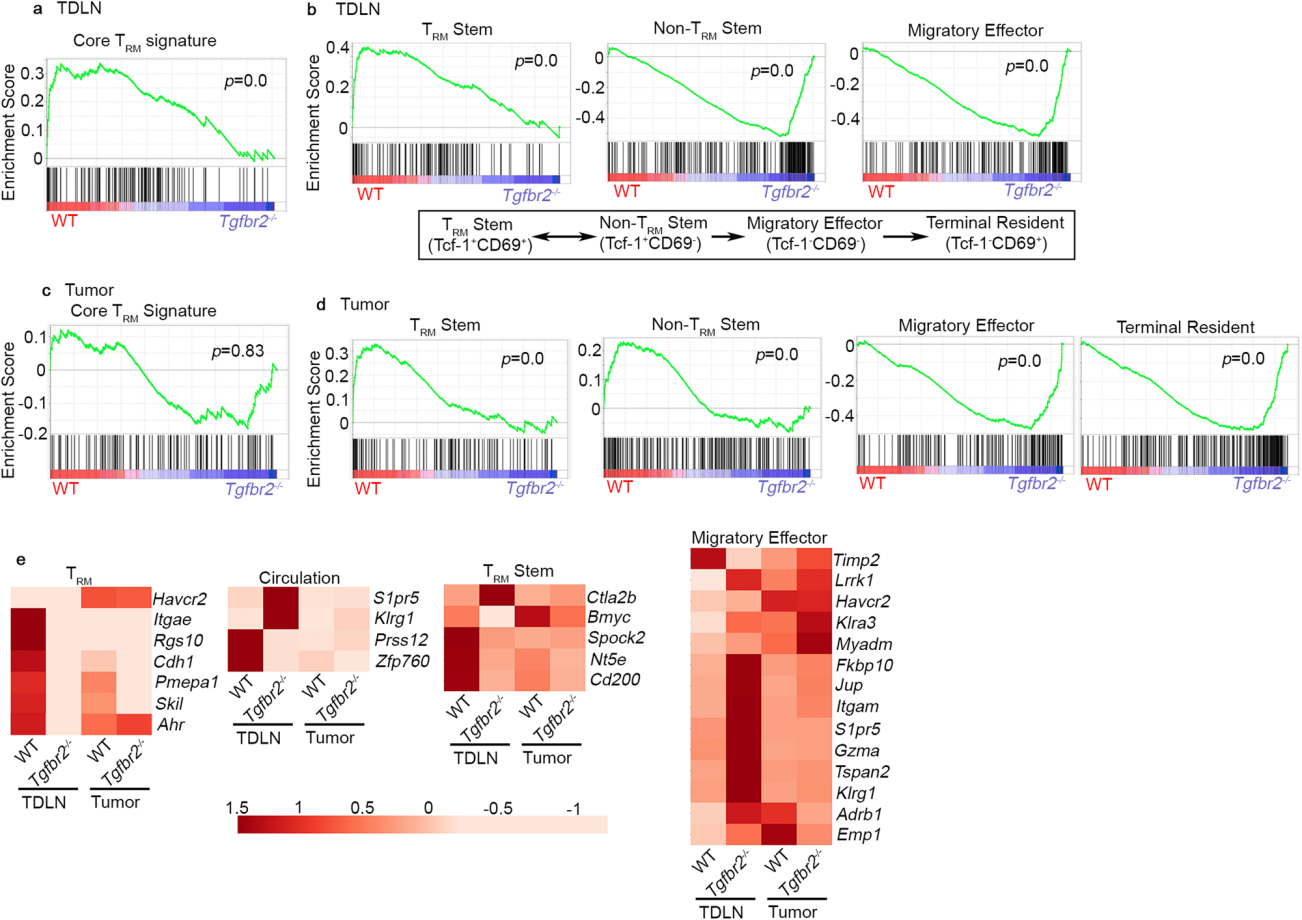
Enhanced differentiation from T_RM_ stem to non-T_RM_ stem in TDLN and accumulation of migratory effectors in tumor for *Tgfbr2*^*−/−*^ Pmel-1 T cells. Bulk RNA-seq was performed on sorted Pmel-1 T cells isolated from TDLN and tumor. GSEA (gene set enrichment analysis) results are shown. Red, WT and violet, *Tgfbr2*^*−/−*^. **a** Core T_RM_ signature enrichment in TDLN samples. **b** The signatures of exhuasted CD8^+^ subsets in TDLN samples. Published differentiation pathway is illustrated in bottom panel. **c** Core T_RM_ signature enrichment in tumor samples. **d** The signatures of exhuasted CD8^+^ subsets in tumor samples. **e** Heatmap of differentially expressed signature genes. All groups contain biological independent duplicates.

To further confirm the T_RM_ identity of stem-like T cells in TDLNs, we FACS sorted WT and *Tgfbr2*^*−/−*^ Pmel-1 T cells from TDLNs and tumors after vaccination. To eliminate the complication introduced by cell migration and focus on immediate local response induced by tumor vaccine, all samples were obtained from FTY720 treated animals. When WT and *Tgfbr2*^*−/−*^ T cells from TDLNs were compared, core T_RM_ signature^49^ was significantly enriched in WT samples (Fig. 9a). In contrast, T_RM_ signature was not significantly enriched when WT and *Tgfbr2*^*−/−*^ TIL samples were compared (Fig. 9c). Specifically, the expression of a collection of T_RM_-associated genes, including *Itgae*, *Rgs10*, *Cdh1*, *Pmepa1*, *Skil*, and *Ahr* was substantially reduced while the expression of circulating T cell signature genes *S1pr5* and *Klrg1* was enhanced in *Tgfbr2*^*−/−*^ TDLN samples (Fig. 9e). Consistently, the expression of a panel of cell adhesion/cytoskeleton-related genes was differentiated between WT vs *Tgfbr2*^*−/−*^ TDLN samples while showing similar patterns of expression in WT vs *Tgfbr2*^*−/−*^ TIL samples (Fig. S8c). These results further validate that T_RM_/cell migration/ movement-associated genes represent the key difference between TDLN WT vs *Tgfbr2*^*−/−*^ CD8^+^ T cells.

Taking advantage of the recently established gene signatures of exhausted CD8^+^ T cell subsets from a mouse chronic viral infection model^26^, we found that CD69^+^Tcf-1^+^ (T_RM_ stem) signature was highly enriched in WT over *Tgfbr2*^*−/−*^ samples from TDLN. In contrast, *Tgfbr2*^*−/−*^ TDLN samples were highly enriched for CD69^−^Tcf-1^+^ (non-T_RM_ stem) and CD69^−^Tcf-1^−^ (migratory effector) signatures (Fig. 9b–e and Fig. S8c). Interestingly, this pattern of gene signature enrichment was TDLN-specific, in TIL Pmel-1 T cells, stem T cell signatures (both T_RM_ stem and non-T_RM_ stem) were enriched in WT while effector and terminally exhausted T cell signatures were enriched in *Tgfbr2*^*−/−*^ samples (Fig. 9d), presumably due to the facts that T cell migration from TDLN to tumor was inhibited by FTY720 and enhanced differentiation from stem→effector occurred inside tumor in the absence of TGF-β signaling. These results further support our conclusion that TDLN functions as a powerhouse and continuous migration from TDLN to tumor is required to sustain *Tgfbr2*^*−/−*^ T cell response to tumor vaccine. To be noted, when comparing TDLN vs TIL samples, core T_RM_ gene signature was highly enriched in TIL samples for both WT and *Tgfbr2*^*−/−*^ (Fig. S8a and S8b), consistent with previous findings^49^ and presumably due to the facts that terminally exhausted T cells (i.e., Tcf-1^−^CD69^+^) carry highly enriched T_RM_ signature^26^. Together, transcriptional profiling is largely consistent with our phenotypic analysis that tumor-specific CD8^+^ T cells differentiate into T_RM_ in TDLNs in a TGF-β-dependent manner. In contrast, for TILs, T_RM_ signature may be controlled by both TGF-β-dependent and -independent pathways.

### T_RM_-like tumor-reactive stem-like CD8^+^ T cells can be detected in human cancer patients

**Fig. 10:**
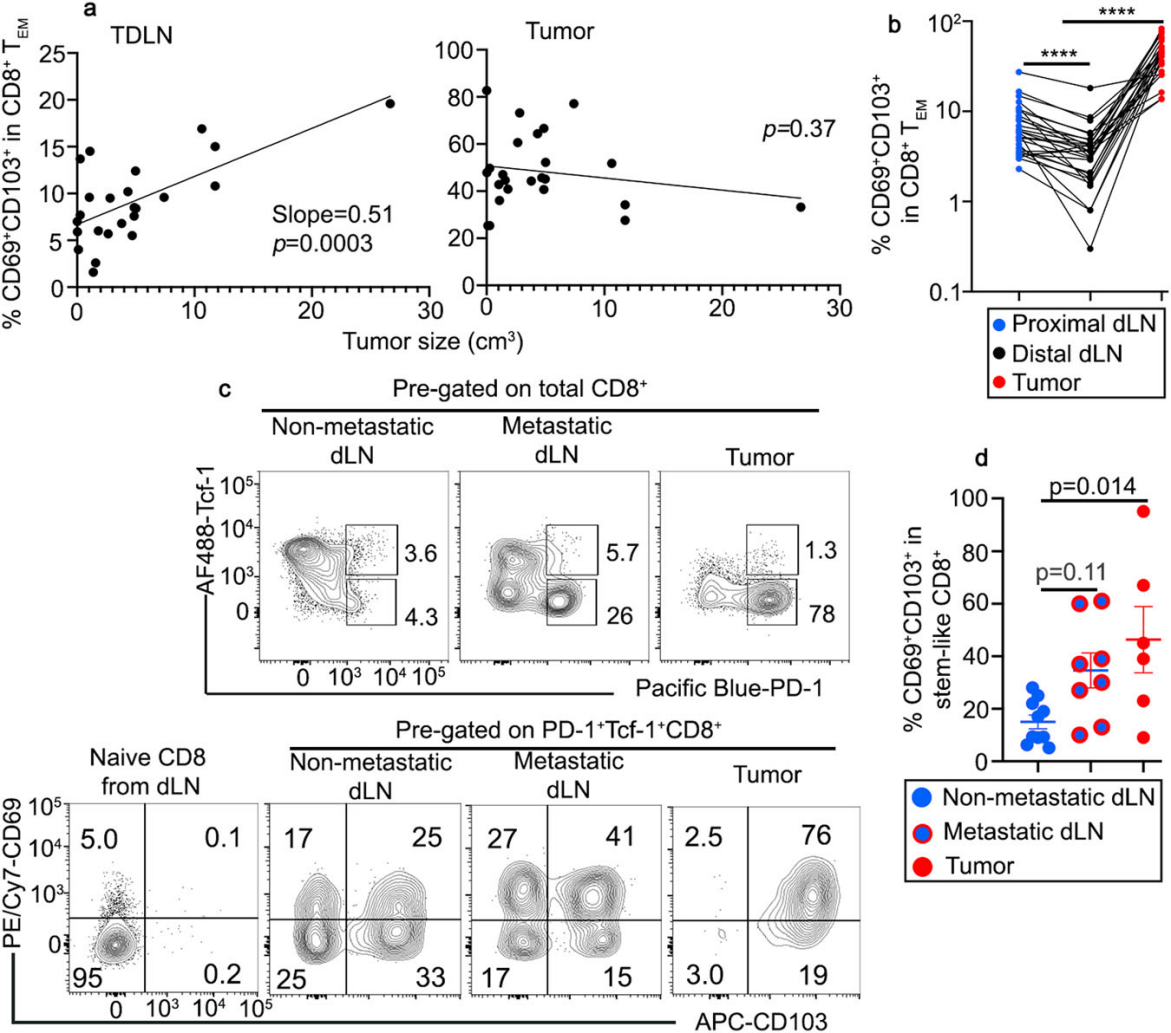
Stem-like CD8^+^ T cells differentiate into T_RM_-like cells in TDLN of human head and neck cancer patients. **a** The association of CD69^+^CD103^+^% in total CD8^+^ T_EM_ isolated from proximal TDLN (left) and tumor (right) are shown (*n* = 24 individual patients). Simple linear regression was performed. **b** The percentage of CD69^+^CD103^+^ cells in total CD8^+^ T_EM_ isolated from proximal TDLN, distal LN and tumor from the same patients (*n* = 28 patients with three samples from each patient). **c** Top, representative FACS profiles of total CD8^+^ T cells isolated from non-metastatic TDLN, metastatic LN and tumor are shown; bottom, compared with TCF-1^+^PD-1^−^CD45RA^+^ naive CD8^+^ T cells, FACS profiles of pre-gated TCF-1^+^PD-1^+^ stem-like CD8 T cells to show the expression of CD69 and CD103. **d** % CD69^+^CD103^+^ cells in pre-gated stem-like CD8^+^ T cells are shown (dLN, *n* = 10, meta LN, *n* = 8 and tumor, *n* = 6). Blue, proximal dLN; black, distal LN; red, tumor; and red circle filled with blue, metastatic dLN. Data are presented as mean ± SEM. Each symbol represents the results from an individual patient. ****p < 0.0001 in **b** are calculated by paired Student *t*-test and indicated *p* values in **d** are calculated by Ordinary one-way ANOVA with multi-comparison posttest. Two-sided tests were used. Source data are provided as a Source data file.

To explore whether our findings in mouse models can be extended to human cancer patients, we focused on a cohort of patients with head and neck squamous cell carcinoma (HNSCC). Indeed, we could detect a clear subset of CD69^+^CD103^+^ T_RM_-like cells in bulk CD8^+^ T_EM_s isolated from the proximal TDLNs (close to the tumor). Importantly, the percentage of T_RM_-like cells was positively correlated with the size of the tumor (Fig. 10a left). This finding is largely consistent with our results in mouse melanoma model (Fig. 4k). As expected for mucosal tumor, we could detect a significant number of CD69^+^CD103^+^ T_RM_-like cells in bulk CD8^+^ T_EM_s isolated from the tumors. However, tumor CD69^+^CD103^+^ T_RM_-like cells were not correlated with tumor size (Fig. 10a right). When comparing proximal TDLN (close to the tumor) vs distal LN (away from the tumor in the same region) isolated from the same patient, we consistently detected more T_RM_-like cells in proximal TDLN, which is in line with our findings that tumor antigen is required for the establishment of T_RM_ program.

Next, we used the expression of PD-1 as a surrogate marker to define tumor-reactive T cells in our samples. As shown in Fig. 10c upper row, compared with non-metastatic TDLNs, we could detect a substantial increase of TCF-1^-^PD-1^+^ CD8^+^ T cells in both metastatic LN and tumor, likely representing non-stem tumor-reactive CD8^+^ T cells (both effectors and terminally exhausted cells). This result further validated PD-1 as a reliable marker to define tumor-reactive T cells in our system. When focusing on TCF-1^+^PD-1^+^ subset (i.e., stem-like tumor-reactive CD8^+^ T cells), we could clearly detect the expression of T_RM_ markers CD103 and CD69 in all samples, including non-metastatic TDLNs (Fig. 10c lower row and 10d). We did observe enhanced T_RM_ marker expression in metastatic TDLN and tumor, presumably due to T_RM_-prone microenvironment established by mucosal tumor cells (Fig. 10c, d). Together, we have demonstrated that tumor-reactive stem-like CD8^+^ T cells can differentiate into T_RM_s in human TDLNs.

## Discussion

Here, we have demonstrated that TDLNs function as a reservoir for tumor-specific stem-like CD8^+^ T cells to reside. A large portion of stem-like T cells differentiate into T_RM_ in a tumor antigen-, CD4-, and TGF-β-dependent manner in TDLNs. Importantly, loss of T_RM_ identity is required for the migration from TDLN to tumor and efficient response to tumor vaccine. For *Tgfbr2*^*−/−*^ stem-like T cells, defective T_RM_ differentiation in TDLN leads to enhanced stem→effector differentiation, elevated and sustained response to tumor vaccine. Importantly, our findings emphasize the two unique features of TDLNs in tumor immunotherapies, i.e., (1) TDLNs function as a reservoir to host stem-like T cells and (2) TDLNs function as a trap to limit the active migration/differentiation of stem-like T cells. Similar to tumor settings, we have recently demonstrated that TGF-β promotes the retention of stem-like CD8^+^ T cells inside lymphoid follicles during chronic viral infection^50,51^. Thus, the TGF-β-dependent lymphoid tissue residency program is not tumor-specific and may represent a universal feature for Tcf-1^+^ CD8^+^ T cells.

A large body of evidence has demonstrated that T_RM_ phenotype of tumor-infiltrating T cells is often associated with improved tumor control and better outcomes^41^. Considering the facts that TGF-β usually promotes T_RM_ differentiation and maintenance, it is challenging to completely explain why TGF-β inhibitors/blockers can synergize with tumor immunotherapies to improve anti-tumor immunity. Our results provide another perspective that the positive correlation of T_RM_ with tumor control may be a tumor-specific observation. TDLN-hosted T_RM_ is negatively associated with direct tumor killing. TDLN-targeted TGF-β blocking strategy may have the potential to be developed into a “universal adjuvant” for tumor vaccine. Further, our results imply that T_RM_ program may have a tissue/organ-specific component in terms of TGF-β dependency.

In contrast to previous findings that tumor vaccine promotes tumor control in WT mice, we did not find detectable delay of tumor progression in vaccinated WT Pmel-1 recipient mice. We believe that the difference is due to the time when tumor vaccine is administrated. In previous research, peptide vaccine is often given when tumor is palpable^9^. In contrast, we administrated tumor vaccine when tumor size reached around 300 mm^3^. Indeed, when comparing early- vs late-stage TDLNs, we found that T_RM_ marker CD69 expression was tightly associated with tumor size. This finding suggests that T_RM_ differentiation is a tumor-stage dependent feature and provides an explanation why early vaccine can boost anti-tumor immunity in WT cells when tissue residency is not fully established in TDLNs.

CD103 is another prominent TGF-β-dependent T_RM_ marker, especially for mucosal T_RM_s. In our system, even though the expression of CD103 was largely consistent with a T_RM_ marker, alternative explanation could not be completely ruled out. CD103 expression was not always associated with CD69 expression. For example, CD103^+^CD69^-^ stem-like T cells could be easily identified in the spleen or early stage TDLN. It has been suggested that CD103^+^CD8^+^ T cells represent a tolerant T cell subset and express Foxp3 at RNA level in a similar B16 tumor model^52^. Alternatively, CD103^+^CD69^−^ cells may represent an intermediate stage of T_RM_ differentiation, similar to the observation of small intestine intra-epithelia lymphocyte (IEL) T_RM_ differentiation at an early stage after viral infection^53^. The difference between CD103^+^CD69^−^ vs CD103^+^CD69^+^ stem-like T cells warrants future investigation. Interestingly, CD4 help is required for CD103, but not CD69 expression on stem-like T cells in TDLN. As TGF-β is required for CD103 induction, this finding is highly consistent with a previous publication showing that CD4 T cell-produced cytokine TGF-β1 suppresses anti-tumor immunity^54^. It is important to elucidate the functions of CD4-derived TGF-β in tumor vaccine settings in the future. Together, we have established that stem-like CD8^+^ T cells differentiate into TDLN-resident T cells in a CD4 help, TGF-β, and tumor antigen-dependent manner. Loss of TDLN residency is required for efficient response to tumor vaccine and differentiate into migratory effectors, which may represent another highly regulated step to be targeted for tumor immunotherapy.

## Methods

### Patient cohorts

Primary tumors, blood and draining lymph nodes from 35 patients with HNSCC were obtained at the Xiangya Hospital from September 2021 to December 2021. The pathological tumor-node-metastasis (TNM) stage was determined according to the American Joint Committee on Cancer (AJCC) 8th edition^55^. The patients’ clinical parameters, such as age, sex, T stage, lymph node metastasis, clinical stage, and tumor size were recorded in detail. Tumor volume was calculated by the modified ellipsoidal formula based on the imagological results (MRI or CT): *V* = π/6 (height × length × width)^56^. Patient exclusion criteria were as follows: the presence of autoimmune disease, human immunodeficiency virus or hepatitis B/C infection, active TB, history of radiotherapy or chemotherapy, and primary HNSCC with other malignancies. The study was approved by the Research Ethics Committee of Central South University, Changsha, China (No. 202108351). Written informed consent was obtained from all patients before surgery. Our reporting complies to the STROBE guidelines.

Human TDLNs, including both proximal and distal LNs to the tumor, belong to the lymph catchment area of the tumor^57^. Specifically, the II/III level of cervical lymph nodes was defined as proximal TDLNs, while the IV level of cervical lymph nodes was defined as distal TDLNs^58^.

### Mice

C57BL/6J (B6, Jax#000664) WT and Pmel-1 TCR transgenic mice (B6.Cg-Thy1^a^/Cy Tg (TcraTcrb) 8Rest/J, Jax#005023) were obtained from the Jackson Laboratory. *Tgfbr2*^f/f^ dLck-cre OT-1 mice were described before^42,59^. *Tgfbr2*^f/f^ mice were originally from S. Karlsson^60^ and dLck-cre mice were originally from N. Killeen^61^. OT-1 mice were originally from Dr. Michael J. Bevan (University of Washington). All mice were housed at our specific pathogen-free animal facilities with 12 h light/dark cycle and 25 °C room temperature at the University of Texas Health at San Antonio (San Antonio, TX). All experimental mice have been backcrossed to C57BL/6 background for more than 12 generations. Both male and female mice are used. All mice are used at 6–18 weeks of age. All experimental animals were euthanized by CO_2_ anesthesia. All experiments were done in accordance with the University of Texas Health Science Center at San Antonio Institutional Animal Care and Use Committee guidelines (protocol number 20180053AR).

### Tumor cell lines

C57BL/6 derived melanoma lines B16F10 and B16-OVA were generous gifts from Dr. Tyler Curiel (UT Health San Antonio). MC38-GP_33_ was a generous gift from Dr. Ananda Goldrath (UCSD)^40^. Both lines were maintained in DMEM complete media (10% FBS + 1% L-glutamine, +1% pen/strep + 0.1 mM non-essential amino acids) at 37 °C in 5% CO_2_. All cell culture medium and supplements were purchased from Invitrogen.

### Naive T cell isolation and adoptive transfer

Naive CD8^+^ T cells (OT-1 or Pmel-1) were isolated from pooled spleen and lymph nodes using MojoSort^TM^ mouse CD8 T cell isolation kit (BioLegend) following manufacturer’s instruction. During the first step of biotin antibody cocktail incubation, biotin-αCD44 (IM7, BioLegend) was added to label and deplete effector and memory T cells. Isolated naive CD8^+^ T cells were numerated, 1:1 mixed when indicated, 10^5^ cells adoptively transferred into each sex-matched unmanipulated B6 recipient via an i.v. route.

### Tumor inoculation and immunotherapies

One day after naive CD8^+^ T cell transfer, the flank of mice was shaved and B16F10 or B16-OVA cells (2.5–3 × 10^5^) were mixed with Matrigel (Corning, final concentration 5 mg/ml) and injected subcutaneously (s.c.). Tumor volumes were estimated by measuring the tumor size in three dimensions (length, width, and height) using a caliper. The tumor volume was calculated according to the formula (π/6 × length × width × height). Mice were sacrificed at the indicated time points or when the estimated tumor volume reached >1000 mm^3^ (endpoint) and the weight of the excised tumor mass was determined.

For tumor vaccination, mice were injected subcutaneously (beside the tumor) with 50 μg/mouse poly(I:C) (Invivogen) together with antigenic peptides (for Pmel-1, gp100_25-33_; for OT-1, OVA_257-264_) at 10 μg/mouse (peptides were purchased from Anaspec). The control (CON) group mice were injected with PBS in same volume.

For checkpoint blockade, mice were injected with rat anti-mouse PD-L1 or isotype control (BioXcell) (200 μg per injection, i.p.) once every 4 days for a total of three injections.

Anti-IFNAR (BioXcell) (500 μg/injection, i.p.) was given twice when indicated. The first time was given together with tumor vaccine and the second injection was administrated 2 days later.

### FTY720 treatment

FTY720 (ENZO Life Sciences), stock solution (4 mg/mL in DMSO) was diluted to 100 μg/mL in double distilled water (dd water) directly before administration and 25 μg/mouse were applied daily by oral gavage for the duration of the experiment. Water containing same concentration of DMSO was used as control.

### Lymphocyte isolation

Blood, spleen, TDLN (inguinal LN on tumor side), NDLN (inguinal LN opposite tumor side, superficial cervical LN, axillary LN), and tumor were isolated. CD8^+^ T cells from spleen and LN were obtained by mashing through a 100 μm nylon cell strainer (BD Falcon). Red blood cells were lysed with a hypotonic Ammonium-Chloride-Potassium (ACK) buffer (prepared in the lab). For TIL isolation, tumors were excised and digested with 1 mg/mL collagenase B (Roche) and 0.02 mg/mL DNaseI (D5025 from Sigma) at 37 °C for 45 min. Digested tumors were mashed through 70 μm filters. Single-cell suspensions were then resuspended in RPMI1640 complete media (10% FBS + 1% L-glutamine, +1% pen/strep + 0.1 mM non-essential amino acids) for flow cytometry.

### Antibodies and flow cytometry

For mouse samples, fluorescence dye-labeled antibodies specific for PD-1 (J43), CD8β (H35-17.2), Granzyme A (GzA-3G8.5), Granzyme B (GB11), CD45.1 (A20), CD45.2 (104), CD8α (53-6.7), CD69 (H1.2F3), CD103 (2E7), CD39 (24DMS1), CD101 (Moushi101), CX3CR1 (SA011F11), Slamf6 (330-AJ) were purchased from eBioscience, BioLegend, Invitrogen, and Tonbo. Anti-CD16/32 (2.4G2) was produced in the lab and used in all FACS staining as FcR blocker. For human samples, fluorescence dye-labeled antibodies specific for PD-1 (EH12.2H7), CD8 (SK1), CD69 (FN50), CD103 (Ber-ACT8), CD45RA (HI100) were purchased from BioLegend. Intranuclear staining for Tcf-1 was performed using a Foxp3 staining buffer set (Tonbo bioscience) and stained with anti-Tcf-1 (C63D9, Cell Signaling). Intracellular staining for Granzyme A and Granzyme B was performed using permeabilization buffer (Invitrogen) following fixation. For intracellular cytokine staining, freshly isolated lymphocytes from tumor were cultured in complete RPMI in the presence of Golgi STOP (BD) with αCD3 (1 μg/ml, 2C11, BioXcell) +αCD28 (1 μg/ml, E18, BioLegend) for 4 h. Stimulated cells were surface stained, fixed, permeablized and intracellular stained by anti-IFN-γ antibody (XMG1.2, BioLegend) and anti-TNF antibody (MP6-XT22, BioLegend). Ghost Dye^TM^ Violet 510 (Tonbo Bioscience) was used to identify live cells. For some tumor samples, fluorescent counting beads (AccuCount Fluorescent Particles from Spherotech) were added before analysis to calculate the number of donor Pmel-1 CD8^+^ T cells. Washed and fixed samples were collected by BD LSRII or BD FACSCelesta using BD FACS Diva software, and analyzed by FlowJO (TreeStar) software.

### Gene expression profiling

Day 4 after tumor vaccine, TDLNs and tumors containing WT and *Tgfbr2*^*−/−*^ Pmel-1 T cells were dissected. To limit T cell migration, FTY720 was administrated. Donor Pmel-1 T cells were FACS sorted based on CD8, CD45.1, and CD45.2. RNA was extracted from sorted cells using the Quick-RNA™ MiniPrep, according to manufacturer’s instructions (Zymo Research). RNA-seq analysis was performed by Novogene.

### Statistical analysis

Ordinary one-way ANOVA, Mantel–Cox, or Student *t*-test from Prism 9 was used.

### Reporting summary

Further information on research design is available in the Nature Research Reporting Summary linked to this article.

## Supplementary information

Supplementary Information

Reporting Summary

## Data Availability

Original flow cytometry data supporting the findings are available from the corresponding author upon request. The sequencing data that supporting the findings of this study have been deposited to NCBI and can be accessed by GSE176525. Source data are provided with this paper.

## Source data

Source Data
