# Supplementary material for: TGF-β-dependent lymphoid tissue residency of stem-like T cells limits response to tumor vaccine
Source: Nat Commun. 2022 Oct 13;13:6043. doi: 10.1038/s41467-022-33768-x (PMC9562983; doi:10.1038/s41467-022-33768-x)
Supplement: Supplementary file 1 — Supplementary Information [file 41467_2022_33768_MOESM1_ESM.pdf]

Supplementary Information

TGF-β-dependent Lymphoid Tissue Residency of Stem-like T cells Limits Response to Tumor Vaccine

Guo Li[1,2,3,4], Saranya Srinivasan[1#], Liwen Wang[1,5#], Chaoyu Ma[1], Kai Guo[2,9], Wenhao Xiao[2], Wei Liao[1,6,10], Shruti Mishra[1,11], Xin Zhang[2,3,4,7], Yuanzheng Qiu[2,3,4,7], Qianjin Lu[6,8], Yong Liu[2,3,4,7*] and Nu Zhang[1*]

Supplementary Figures

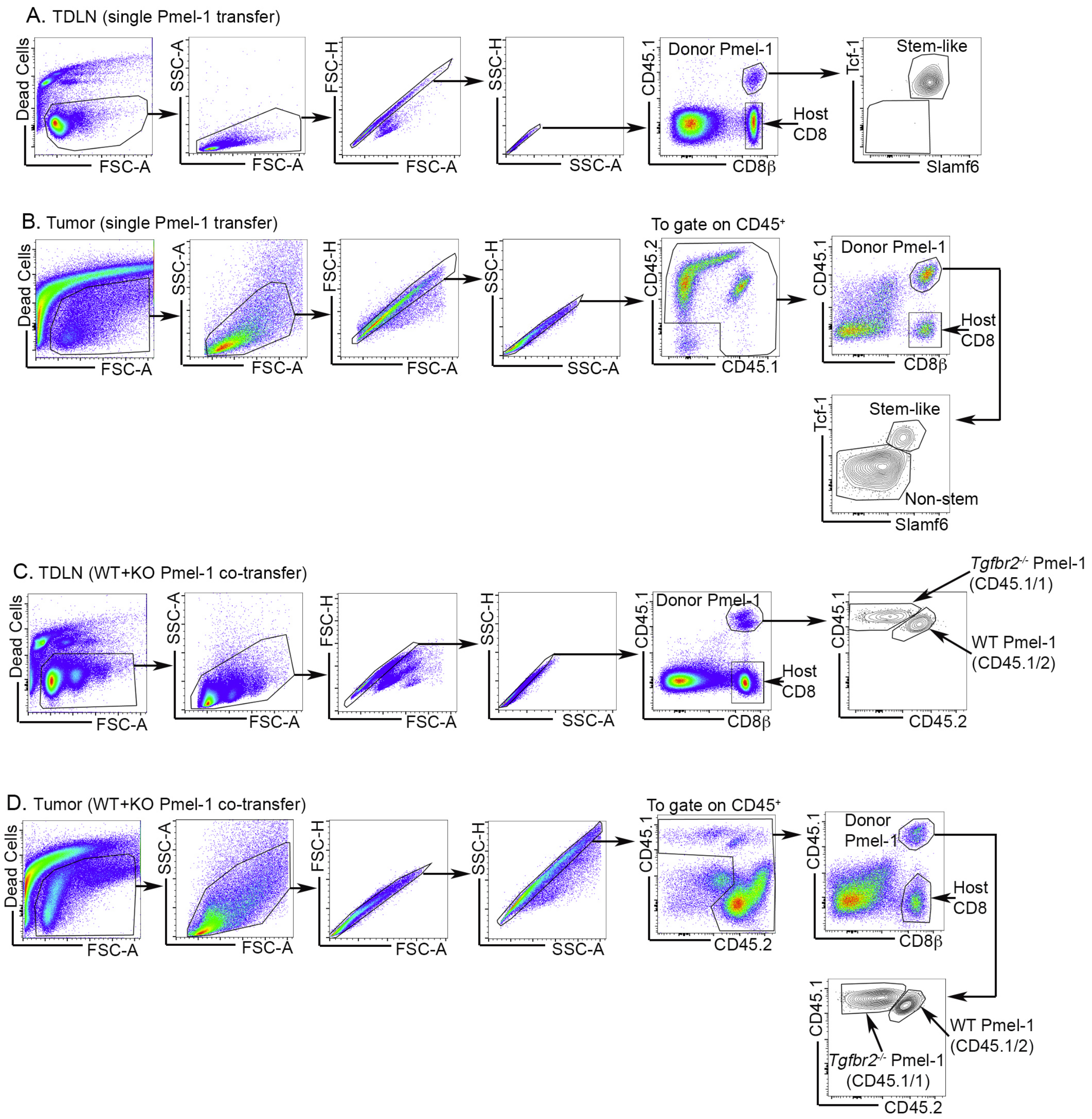

Supplementary Figure 1. FACS gating strategies. For single Pmel-1 donor T cell transfer experiments, gating strategies for TDLN (**A**, used in Fig. 3, 4, 5, 8, S2, S3, S4 and S7) and tumor (**B,** used in Fig. 2, 3, 4, 8, S2, S3 and S7) are shown. For WT (wild type) and *Tgfbr2*$^{-/-}$ Pmel-1 co-transfer experiments, gating strategies for TDLN (**C,** used in Fig. 5, 6, 7, S5 and S6) and tumor (**D,** used in Fig. 6, 7, S5 and S6) are shown.

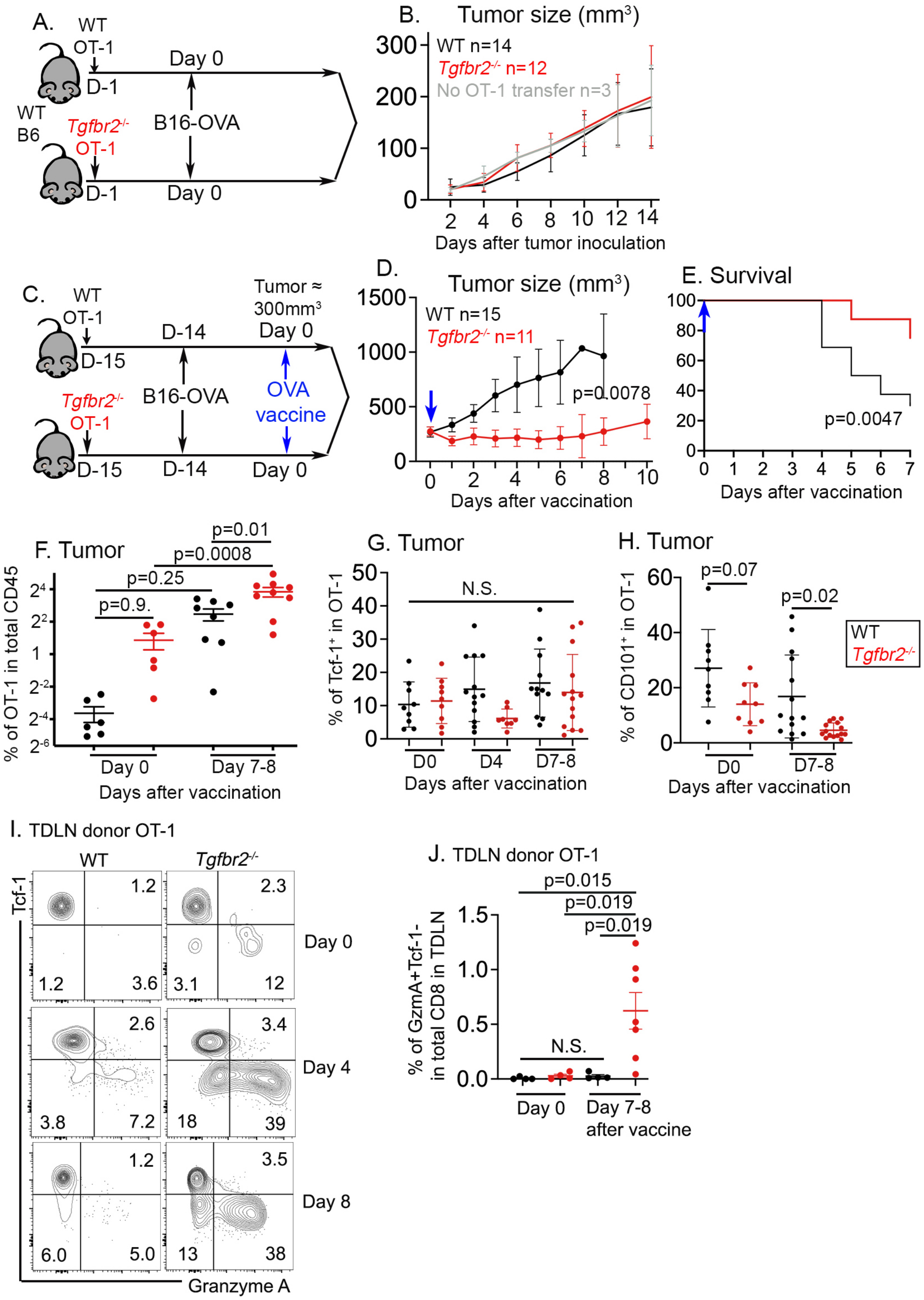

Supplementary Figure 2. *Tgfbr2$^{-/-}$* OT-1 T cells synergize with OVA peptide vaccination to control B16-OVA. (**A**) and (**B**), OT-1 transfer alone or No OT-1 control in grey. (**A**) Schematics; (**B**) Tumor growth. (**C**) to (**E**), OT-1+OVA vaccine. (**C**) Schematics; (**D**) Tumor growth and (**E**) Survival curve after vaccination are shown. Black, WT OT-1 recipients, red, *Tgfbr2$^{-/-}$* recipients and grey, no T cell transfer. For (D) and (E),

WT, n=15 and *Tgfbr2*$^{-/-}$, n=11. (**F**) The percentage of OT-1 T cells in total CD45^{+} tumor infiltrating cells is shown (WT, n=6, *Tgfbr2*$^{-/-}$, n=6, WT+Vaccine, n=8 and *Tgfbr2*$^{-/-}$, n=9). (**G**) The percentage of Tcf-1^{+} subset in tumor infiltrating OT-1 T cells is shown (WT d0, n=9, *Tgfbr2*$^{-/-}$ d0, n=9, WT d4, n=13, *Tgfbr2*$^{-/-}$ d4, n=8, WT d8, n=12 and *Tgfbr2*$^{-/-}$ d8, n=14). (**H**) The percentage of CD101^{+} subset in tumor infiltrating OT-1 T cells is shown(WT, n=9, *Tgfbr2*$^{-/-}$, n=9, WT+Vaccine, n=14 and *Tgfbr2*$^{-/-}$, n=15). (**I**) Representative FACS profiles of TDLN OT-1 T cells are shown (WT, n=4, *Tgfbr2*$^{-/-}$, n=4, WT+Vaccine, n=4 and *Tgfbr2*$^{-/-}$, n=7). (**J**) The percentage of granzyme A^{+}Tcf-1^{-} cells in TDLN OT-1 T cells are shown. Each symbol in (F to J) represents the results from an individual mouse. Black symbols, WT and red symbols, *Tgfbr2*$^{-/-}$. Data are presented as mean±SEM. Pooled results from 2-3 independent experiments are shown. N.S., not significant (p>0.05) and indicated p values are calculated by two tailed Wilcoxon test (D), Log-rank Mantel-Cox test (E) or Ordinary one-way ANOVA with Tukey's multi-comparison posttest (F to H, and J). Two sided tests were used. Source data are provided as a Source Data file.

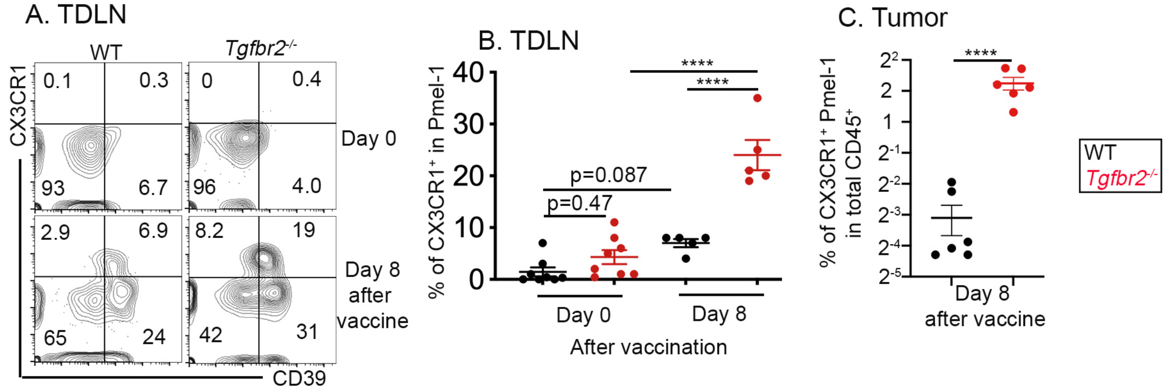

Supplementary Figure 3. Tumor vaccine boosts the differentiation of CX3CR1⁺ migratory effectors in TDLN and tumor. Similar experimental setup as in Fig. 2A. (**A**) Representative FACS profiles of donor Pmel-1 T cells in TDLN before and after tumor vaccine are shown. (**B**) The percentage of CX3CR1⁺ effectors in Pmel-1 T cells from TDLN is shown (WT, n=8, *Tgfbr2⁻/⁻*, n=8, WT+Vaccine, n=5 and *Tgfbr2⁻/⁻*, n=5). (**C**) The percentage of CX3CR1⁺ Pmel-1 T cells in total CD45⁺ tumor infiltrating cells after tumor vaccine is shown (n=6 for both WT and *Tgfbr2⁻/⁻*). Pooled results from 2 independent experiments are shown. Each symbol in (B) and (C) represents the results from an individual mouse. Black symbols, WT and red, *Tgfbr2⁻/⁻*. Data are presented as mean±SEM. Indicated p values and ****, $p<0.0001$ are calculated by Ordinary one-way ANOVA with Tukey's multi-comparison posttest (B) or two-tailed unpaired Student *t*-test (C). Two sided tests were used. Source data are provided as a Source Data file.

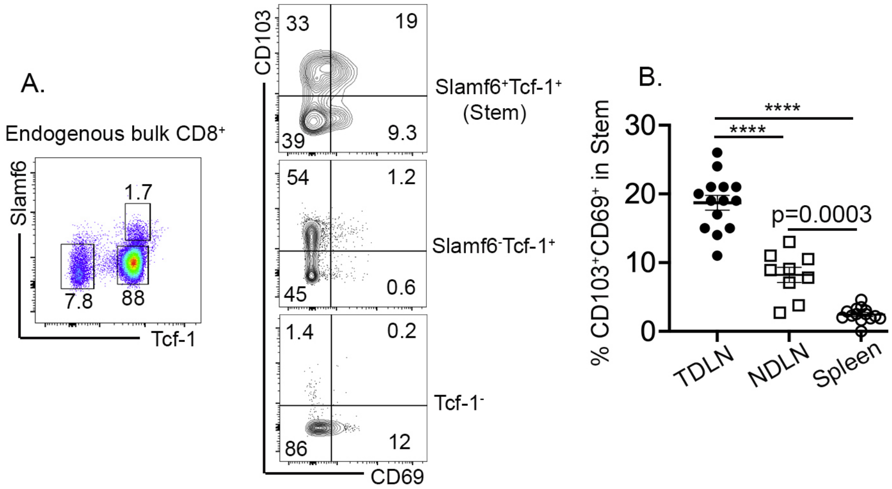

Supplementary Figure 4. Stem-like endogenous polyclonal CD8$^+$ T cells differentiate into T$_{RM}$ in TDLN. Same experimental setup as in Fig. 4a. (**A**) Left, representative gating strategy on endogenous bulk CD8$^+$ T cells isolated from TDLN; Right, representative FACS profiles of pre-gated endogenous CD8$^+$ T cells to show T$_{RM}$ phenotype. (**B**) The percentage of CD69$^+$CD103$^+$ subset in endogenous stem-like CD8$^+$ T cells isolated from different lymphoid organs (TDLN, n=14, NDLN, n=9 and spleen, n=14). Data are presented as mean±SEM. Each symbol in (B) represents the results from an individual mouse. Filled circle, TDLN, empty square, NDLN and empty circle, spleen. Pooled results from 3 independent experiments are shown. Indicated *p* value and ****, *p*<0.0001 are calculated by Ordinary one-way ANOVA with Tukey's multi-comparison posttest. Two sided tests were used. Source data are provided as a Source Data file.

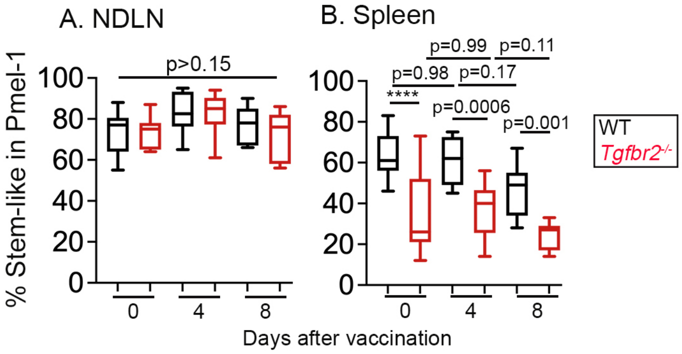

Supplementary Figure 5. Stem-like T cell subset in other lymphoid organs after tumor vaccination. Same experimental setup as in Fig. 7. The percentage of stem-like subset in donor Pmel-1 T cells isolated from NDLN (**A**) and spleen (**B**) at different time points after vaccination are shown. For (A), WT d0, n= 9, *Tgfbr2*$^{-/-}$ d0, n=9, WT d4, n= 10, *Tgfbr2*$^{-/-}$ d4, n=10, WT d8, n= 11 and *Tgfbr2*$^{-/-}$ d8, n=11. For (B), WT d0, n= 11, *Tgfbr2*$^{-/-}$ d0, n=11, WT d4, n= 13, *Tgfbr2*$^{-/-}$ d4, n=13, WT d8, n= 11 and *Tgfbr2*$^{-/-}$ d8, n=11. Data are presented as mean±SEM. Pooled results from 3 independent experiments are shown. Black, WT and red, *Tgfbr2*$^{-/-}$. Indicated *p* values and ****, *p*<0.0001 are calculated by Ordinary one-way ANOVA with Tukey's multi-comparison posttest. Two sided tests were used. Source data are provided as a Source Data file.

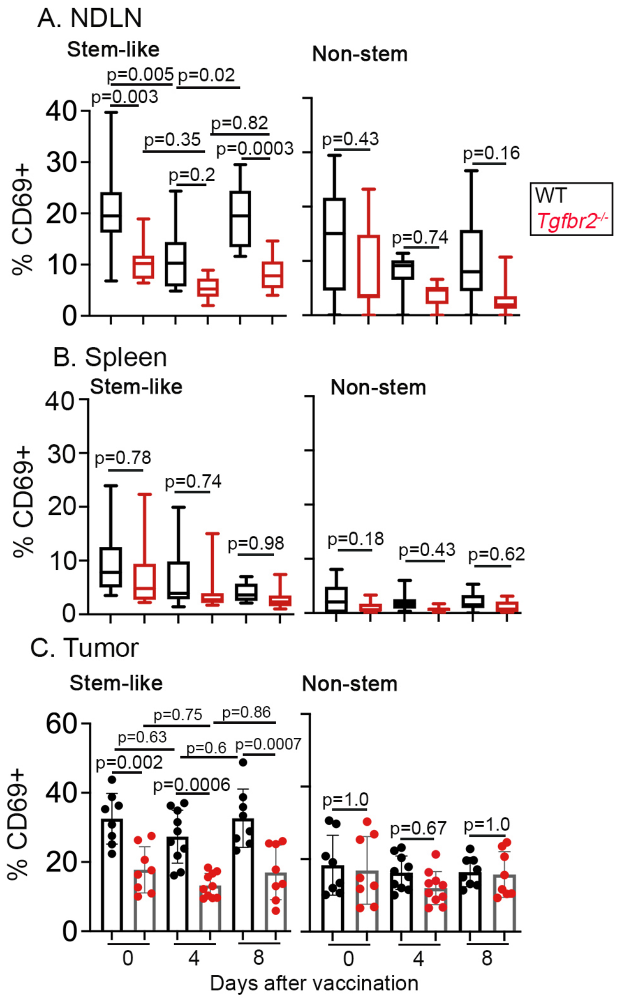

Supplementary Figure 6. The alteration of tissue residency after tumor vaccination. Same experimental setup as in Fig. 7. (**A**) The percentage of $CD69^+$ cells in stem-like (Left) and non-stem (Right) Pmel-1 T cells isolated from NDLN are shown. For stem-like, WT d0, n= 9, *Tgfbr2*$^{-/-}$ d0, n=9, WT d4, n= 10, *Tgfbr2*$^{-/-}$ d4, n=10, WT d8, n= 11 and *Tgfbr2*$^{-/-}$ d8, n=11. For non-stem, WT d0, n= 9, *Tgfbr2*$^{-/-}$ d0, n=9, WT d4, n= 10, *Tgfbr2*$^{-/-}$ d4, n=10, WT d8, n= 11 and *Tgfbr2*$^{-/-}$ d8, n=11. (**B**) The percentage of $CD69^+$ cells in stem-like (Left) and non-stem (Right) Pmel-1 T cells isolated from spleen are shown. For stem-like, WT d0, n= 11, *Tgfbr2*$^{-/-}$ d0, n=11, WT d4, n= 13, *Tgfbr2*$^{-/-}$ d4, n=13, WT d8, n= 11 and *Tgfbr2*$^{-/-}$ d8, n=11. For non-stem, WT d0, n= 11, *Tgfbr2*$^{-/-}$ d0, n=11, WT d4, n= 13, *Tgfbr2*$^{-/-}$ d4, n=13, WT d8, n= 11 and *Tgfbr2*$^{-/-}$ d8, n=11. (**C**) The percentage of $CD69^+$ cells in stem-like (Left) and non-stem (Right) Pmel-1 T cells isolated from tumors at different time points after vaccination are shown. For stem-like, WT d0, n= 8, *Tgfbr2*$^{-/-}$ d0, n=8, WT d4, n= 10, *Tgfbr2*$^{-/-}$ d4, n=10, WT d8, n= 8 and *Tgfbr2*$^{-/-}$ d8, n=8. For non-stem, WT d0, n= 8, *Tgfbr2*$^{-/-}$ d0, n=8, WT d4, n= 10, *Tgfbr2*$^{-/-}$ d4, n=10, WT d8, n= 8 and *Tgfbr2*$^{-/-}$ d8, n=8.Pooled results from 3 independent experiments are shown. Data are presented as mean±SEM. Each symbol in (C) represents the results from an individual recipient mouse. Black symbols, WT and red symbols, *Tgfbr2*$^{-/-}$. Indicated p values are calculated by Ordinary one-way ANOVA with Tukey's multi-comparison posttest. Two sided tests were used. Source data are provided as a Source Data file.

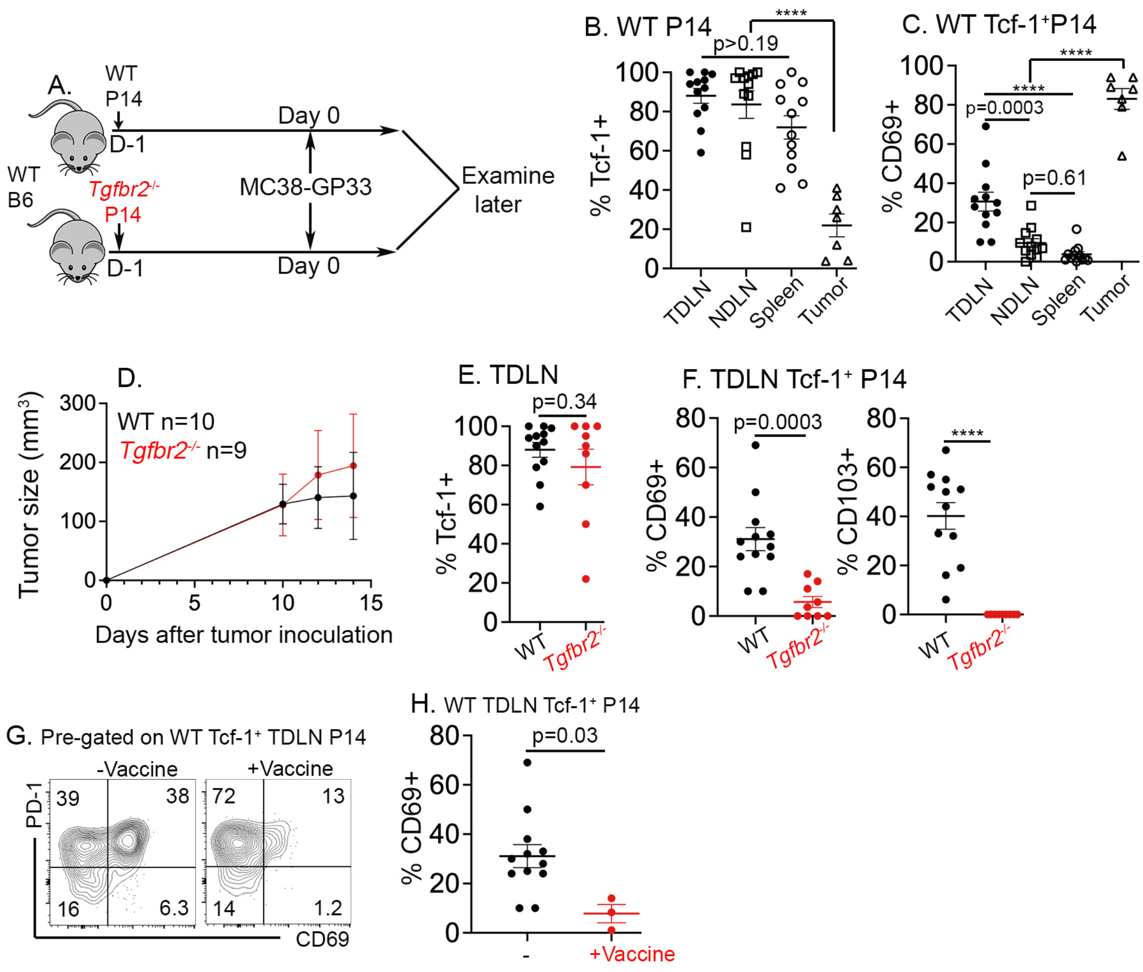

Supplementary Figure 7. Colorectal tumor induces T_{RM} stem-like $CD8^{+}$ T cell differentiation in TDLN. (**A**) Experimental design. (**B**) The percentage of Tcf-1^{+} cells in WT donor P14 T cells are shown. Filled circle, TDLN, empty square, NDLN, empty circle, spleen and empty triangle, tumor. (**C**) The percentage of CD69^{+} cells in WT Tcf-1^{+} P14 T cells are shown. For (B) and (C), TDLN, n=12, NDLN, n=12, Spleen, n=12 and Tumor, n=7. (**D**) Tumor growth (WT, n=10 and *Tgfbr2$^{-/-}$*, n=9). (**E**) The percentage of Tcf-1^{+} cells in WT and *Tgfbr2$^{-/-}$* P14 T cells isolated from TDLN are shown (WT, n=12 and *Tgfbr2$^{-/-}$*, n=9). (**F**) The percentage of CD69^{+} (left) and CD103^{+} (right) in TDLN Tcf-1^{+} P14 T cells are shown (WT, n=12 and *Tgfbr2$^{-/-}$*, n=9). (**G**) Representative FACS of pre-gated TDLN Tcf-1^{+} P14 T cells before and 4 days after vaccination are shown. (**H**) The percentage of CD69^{+} cells in Tcf-1^{+} P14 T cells isolated from TDLN are shown (-Vaccine, n=12 and +Vaccine, n=3). Each symbol in (B to C) and (E, F, and H) represents the results from an individual mouse. For (D) to (F), black symbols, WT and red symbols, *Tgfbr2$^{-/-}$*. For (H), black symbols, no vaccine and red symbols, after vaccine. Data are presented as mean±SEM. Pooled results from 2 independent experiments are shown. Indicated p values and ****, $p<0.0001$ by Ordinary one-way ANOVA with Tukey's multi-comparison posttest. (B and C) or unpaired Student *t*-test (E to H). Two sided tests were used. Source data are provided as a Source Data file.

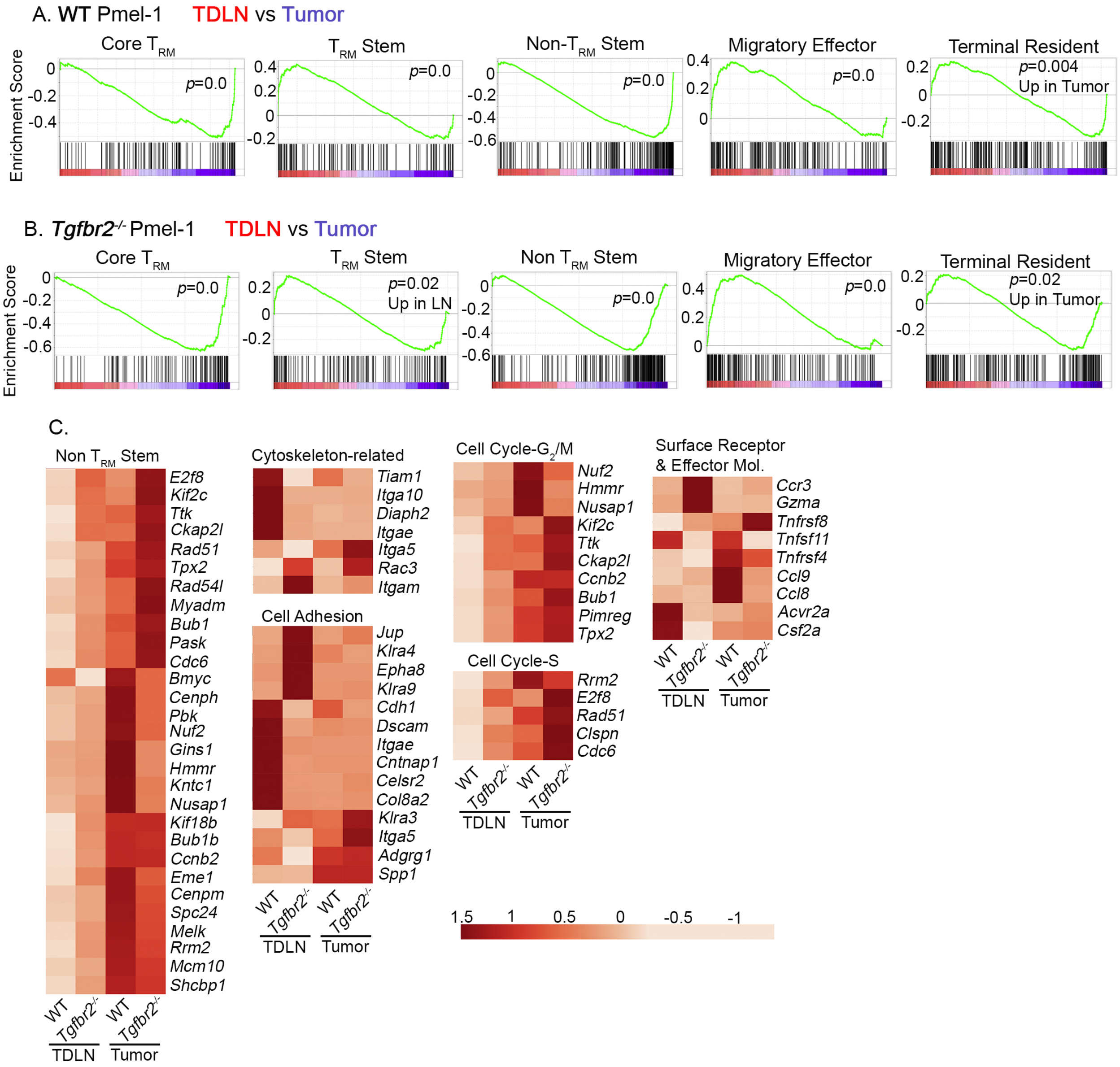

Supplementary Figure 8. Enrichment of T_{RM} signature genes in tumor. GSEA results comparing TDLN (red) vs tumor (violet) samples. (**A**) WT and (**B**) *Tgfbr2*$^{-/-}$ samples (**C**) Heatmap of DEGs for selected signature genes.
