# Supplementary material for: TGF-β-dependent lymphoid tissue residency of stem-like T cells limits response to tumor vaccine
Source: Nat Commun. 2022 Oct 13;13:6043. doi: 10.1038/s41467-022-33768-x (PMC9562983; doi:10.1038/s41467-022-33768-x)
Supplement: Supplementary file 2 — Reporting Summary [file 41467_2022_33768_MOESM2_ESM.pdf]

nature portfolio

Corresponding author(s): Nu Zhang

Last updated by author(s): Sep 6, 2022

Reporting Summary

Nature Portfolio wishes to improve the reproducibility of the work that we publish. This form provides structure for consistency and transparency in reporting. For further information on Nature Portfolio policies, see our Editorial Policies and the Editorial Policy Checklist.

Statistics

For all statistical analyses, confirm that the following items are present in the figure legend, table legend, main text, or Methods section.

n/a	Confirmed	
☐	☒	The exact sample size (n) for each experimental group/condition, given as a discrete number and unit of measurement
☒	☐	A statement on whether measurements were taken from distinct samples or whether the same sample was measured repeatedly
☐	☒	The statistical test(s) used AND whether they are one- or two-sided<br>*Only common tests should be described solely by name; describe more complex techniques in the Methods section.*
☐	☒	A description of all covariates tested
☐	☒	A description of any assumptions or corrections, such as tests of normality and adjustment for multiple comparisons
☐	☒	A full description of the statistical parameters including central tendency (e.g. means) or other basic estimates (e.g. regression coefficient) AND variation (e.g. standard deviation) or associated estimates of uncertainty (e.g. confidence intervals)
☐	☒	For null hypothesis testing, the test statistic (e.g. F, t, r) with confidence intervals, effect sizes, degrees of freedom and P value noted<br>*Give P values as exact values whenever suitable.*
☒	☐	For Bayesian analysis, information on the choice of priors and Markov chain Monte Carlo settings
☒	☐	For hierarchical and complex designs, identification of the appropriate level for tests and full reporting of outcomes
☒	☐	Estimates of effect sizes (e.g. Cohen's d, Pearson's r), indicating how they were calculated

Our web collection on statistics for biologists contains articles on many of the points above.

Software and code

Policy information about availability of computer code

Data collection	FACS Diva for Flow cytometry data collection
Data analysis	FlowJo9 and FlowJo10 for Flow cytometry data analysis. Prism 9 for statistical analysis.

For manuscripts utilizing custom algorithms or software that are central to the research but not yet described in published literature, software must be made available to editors and reviewers. We strongly encourage code deposition in a community repository (e.g. GitHub). See the Nature Portfolio guidelines for submitting code & software for further information.

Data

Policy information about availability of data

All manuscripts must include a data availability statement. This statement should provide the following information, where applicable:

- Accession codes, unique identifiers, or web links for publicly available datasets
- A description of any restrictions on data availability
- For clinical datasets or third party data, please ensure that the statement adheres to our policy

Source data are provided in the online version of the paper. RNAseq results can be accessed by GSE176525. Other data are available from the corresponding author upon reasonable request.

Field-specific reporting

Please select the one below that is the best fit for your research. If you are not sure, read the appropriate sections before making your selection.

- [x] Life sciences
- [ ] Behavioural & social sciences
- [ ] Ecological, evolutionary & environmental sciences

For a reference copy of the document with all sections, see nature.com/documents/nr-reporting-summary-flat.pdf

Life sciences study design

All studies must disclose on these points even when the disclosure is negative.

Sample size	Based on our preliminary experiments, 4-6 mice/group/time point was sufficient to detect difference between groups. Thus, we used 4-6 mice/group/time point. Pooled results from independent experiments are shown in most figures as stated in figure legends.
Data exclusions	When B16 tumor is subcutaneously injected into C57BL/6 mice, we typically observe around 5% recipients not developing tumor regardless of Pmel or OT-1 T cell transfer. These mice are excluded from further manipulation (e.g., tumor vaccine or PD-L1) and analysis.
Replication	At least 2-3 biological independent replications were performed for every experiment. All repeats were successful.
Randomization	For all mouse experiments, the recipient C57BL/6 mice (age and sex matched) were directly purchased from Jax and used after 2-4 weeks housing in our animal facility. Recipient mice with similar tumor size were randomly picked for either control or experimental treatment group. For experiments using human samples, we only included one group of patients without any treatment. As we focused on tumor draining lymph nodes, health control samples were not relevant.
Blinding	Blinding was not possible as all experiments were coordinated by a single investigator.

Reporting for specific materials, systems and methods

We require information from authors about some types of materials, experimental systems and methods used in many studies. Here, indicate whether each material, system or method listed is relevant to your study. If you are not sure if a list item applies to your research, read the appropriate section before selecting a response.

Materials & experimental systems

n/a	Involved in the study	
	x	Antibodies
	x	Eukaryotic cell lines
x		Palaeontology and archaeology
	x	Animals and other organisms
	x	Human research participants
x		Clinical data
x		Dual use research of concern

Methods

n/a	Involved in the study	
x		ChIP-seq
	x	Flow cytometry
x		MRI-based neuroimaging

Antibodies

Antibodies used

Ab Clone# Vendor Cat# Dilution
Pacific Blue-TNF MP6-XT22 Biolegend 506318 1:100
PE/Cy7-IFNg XMG1.2 Biolegend 505826 1:250
AF488-Tcf-1 C63D9 Cell Signaling 6444S 1:150
PE-Granzyme A GzA-3G8.5 Thermofisher 12-5831-82 1:100
PE/Cy7-CD101 Moushi101 Thermofisher 25-1011-82 1:100
PE-CD103 2E7 Biolegend 121406 1:100
BV605-CD103 2E7 Biolegend 121433 1:100
PerCP-Cy5.5-CD69 H1.2-F3 Tonbo 65-0691-U100 1:100
FITC-CD62L MEL-14 Biolegend 104406 0.111111111
PE-CD62L MEL-14 Biolegend 104408 1:100
SB780-CD44 IM7 Thermofisher 78-0441-82 1:100
eFluor450-CD8b H35-17.2 Thermofisher 48-0083-82 1:100
APC-eFluor780-CD8b H35-17.2 Thermofisher 47-0083-82 1:100
APC-eFluor780-CD4 GK1.5 Thermofisher 47-0041-82 1:100
APC-Slamf6 330-AJ Biolegend 134610 1:100
PE/Cy7-PD-1 J43 Thermofisher 25-9985-82 1:100
APC-CD38 90 Thermofisher 17-0381-82 1:100

PE/Cy7-Tim-3 RMT3-23 Biolegend 119716 1:100
SB600-CD45.1 A20 Thermofisher 63-0453-82 1:100
SB780-CD45.2 104 Thermofisher 78-0454-82 1:50
FITC-Granzyme B NGZB Thermofisher 11-8898-82 1:200
Alexa Fluor 647-CX3CR1 SA011F11 Biolegend 149004 1:250
PE-Cy7-CX3CR1 SA011F11 Biolegend 149016 1:100
PE-CD39 24DMS1 Thermofisher 12-0391-82 1:100

Validation	All antibodies involved in the current study are commonly available from commercial vendors. All antibody clones have been validated by the vendors. Validation data are available on the manufacturer's website.

Eukaryotic cell lines

Policy information about cell lines

Cell line source(s)	B16F10 and B16OVA lines are gifts from Dr. Tyler Curiel at UT Health San Antonio. MC38-GP33 line is a gift from Dr. Ananda Goldrath, UCSD.
Authentication	The cells used were not authenticated.
Mycoplasma contamination	Have not tested for mycoplasma contamination
Commonly misidentified lines (See ICLAC register)	No commonly misidentified cell lines were used in this study.

Animals and other organisms

Policy information about studies involving animals; ARRIVE guidelines recommended for reporting animal research

Laboratory animals	C57BL/6J (B6) WT and Pmel-1 TCR transgenic mice (B6.Cg-Thy1a/Cy Tg (TcraTcrb) 8Rest/J) were obtained from the Jackson Laboratory. Tgfbr2f/f dLck-cre OT-1 mice were described before (13, 66). Tgfbr2f/f mice were originally from S. Karlsson (67) and dLck-cre mice were originally from N. Killeen (68). OT-1 mice were originally from Dr. Michael J. Bevan (University of Washington). All mice were housed at our specific pathogen-free animal facilities at the University of Texas Health at San Antonio (San Antonio, TX). All experimental mice have been backcrossed to C57BL/6 background for more than 12 generations. Both male and female mice were used. All mice were used at 6-18 weeks old.
Wild animals	No wild animals were used in this study.
Field-collected samples	No field-collected samples were used in this study.
Ethics oversight	All study protocols have been approved by UT Health San Antonio IACUC.

Note that full information on the approval of the study protocol must also be provided in the manuscript.

Human research participants

Policy information about studies involving human research participants

Population characteristics	All patients are treatment naive. Patients with active TB are excluded. Most recruited patients are males (from age 35 to 73, average age 57.6) and one patient is female (age 78).
Recruitment	Patients shown up at outpatient clinic at Xiangya Hospital are recruited during indicated period. Untreated HNSCC patients without active TB are the only selection criteria. Informed consent was obtained. No special compensation was provided for study participants. We are not aware of any biases that may impact the results.
Ethics oversight	Medical Ethics Committee of Xiangya Hospital, Central South University

Note that full information on the approval of the study protocol must also be provided in the manuscript.

Flow Cytometry

Plots

Confirm that:

- [x] The axis labels state the marker and fluorochrome used (e.g. CD4-FITC).
- [x] The axis scales are clearly visible. Include numbers along axes only for bottom left plot of group (a 'group' is an analysis of identical markers).
- [x] All plots are contour plots with outliers or pseudocolor plots.
- [x] A numerical value for number of cells or percentage (with statistics) is provided.

Methodology

Sample preparation	Single cell suspension from spleen, tumor and LN was incubated with FcR blocker. Tumors were excised and digested with 1 mg/mL collagenase B (Roche) and 0.02 mg/mL DNaseI (D5025 from Sigma) at 37°C for 45min. Digested tumors were mashed through 70 um filters.
Instrument	BD LSRII and BD FACSCelesta
Software	FACS Diva for data collection. FlowJo for data analysis
Cell population abundance	Depending on the times and tissues, our interested cell population (donor-derived Pmel-1 T cells) can be ranged from less than 1% to over 20% of total CD8 T cells.
Gating strategy	Lymphocyte population was gated based on FSC-A and SSC-A. Further, single cells was gated for further analysis based on FSC-A/FSC-H and SSC-A/SSC-H. All samples were further gated on live cells based on Ghost DyeTM Violet 510 (Tonbo bioscience) staining.

☒ Tick this box to confirm that a figure exemplifying the gating strategy is provided in the Supplementary Information.
